# Reversible dioxygen uptake at [Cu_4_] clusters†

**DOI:** 10.1039/d3sc06390a

**Published:** 2024-03-07

**Authors:** Manasseh Kusi Osei, Saber Mirzaei, M. Saeed Mirzaei, Agustin Valles, Raúl Hernández Sánchez

**Affiliations:** a Department of Chemistry, Rice University 6100 Main St. Houston Texas USA raulhs@rice.edu; b Department of Chemistry, University of Pittsburgh 219 Parkman Ave. Pittsburgh Pennsylvania 15260 USA

## Abstract

Dioxygen binding solely through non-covalent interactions is rare. In living systems, dioxygen transport takes place *via* iron or copper-containing biological cofactors. Specifically, a reversible covalent interaction is established when O_2_ binds to the mono or polynuclear metal center. However, O_2_ stabilization in the absence of covalent bond formation is challenging and rarely observed. Here, we demonstrate a unique example of reversible non-covalent binding of dioxygen within the cavity of a well-defined synthetic all-Cu(i) tetracopper cluster.

## Abstract 2

Well-defined systems binding dioxygen solely through non-covalent interactions are rare.

## Graphical abstract

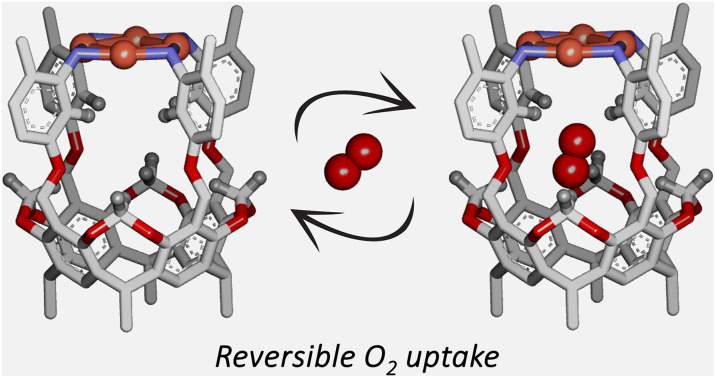

## Introduction

Reversible O_2_ binding is the cornerstone of cellular respiration.^1^ Hemoglobin, myoglobin, hemerythrin, and hemocyanin all serve as O_2_ transporters across all living organisms.^2^ In the process of biological uptake, transport, and delivery of dioxygen, a covalent bond is established between O_2_ and Fe,^3^ or similarly between O_2_ and the Cu_2_ site in hemocyanin,^4^ resulting in charge transfer to form a superoxo or peroxo moiety, respectively. Although, non-covalent interactions have been described to be operative in stabilizing ferric–superoxo intermediates in heme proteins,^5^ these nominally weak contacts are generally challenging to study.

Aside from biological cofactors, numerous studies have reported supramolecular complexes, porous materials, and organic cages capable of binding dioxygen. For instance, metal complexes formed within macrocyclic species, *e.g.*, palladium-bound cyclodextrin,^6^ and Mn-supported calixarene,^7^ lead to peroxo and superoxo moieties, respectively, stabilized inside the macrocycle. Similar occurrences are observed within metal organic frameworks (MOFs), where O_2_ binding to embedded metal sites is used for O_2_/N_2_ separations,^8^ or bond activation cleaving the O–O bond.^9,10^ Last, macrocycles alone are also known to stabilize peroxo species.^11^ However, to our knowledge, non-covalent interactions alone have not been described to stabilize neutral O_2_ in biological metal cofactors or synthetic metal clusters. Here, we describe a polynuclear copper cluster built within a flexible supramolecular scaffold capable of creating a unique pocket binding O_2_ solely through non-covalent interactions.

**Fig. 1:**
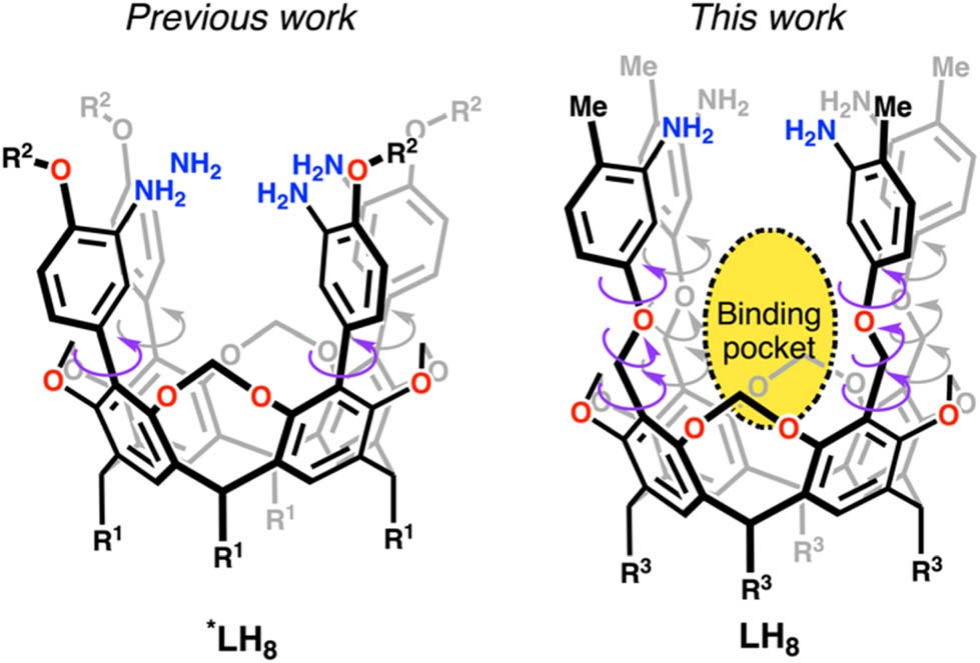
Previous ligand architecture *LH_8_ (R^1^ = *n*-pentyl; R^2^ = Me, Ph, or i-Bu) compared to LH_8_ (R^3^ = *n*-heptyl) reported herein with increased degrees of freedom.

Our group has developed modular amine-based ligands serving as templates for metal cluster formation. Recently, we reported a rigid ligand scaffold that enables the formation of square planar tetranuclear [Cu_4_] clusters (Fig. 1).^12^ Keeping some of the design principles employed before, we decided to increase the degrees of freedom of the ligand scaffold as shown in Fig. 1. Others in the field have adopted similar measures either allowing or restricting the templating ligand's rigidity to alter the cluster reactivity and composition. For example, Betley and coworkers demonstrated that increasing the template's rigidity exchanging the tame ligand backbone (tame = 1,1,1-tris(aminomethyl)ethane),^13–16^ for the tris-amine α-α-α-1,3,5-tris-aminocyclohexane,^17–19^ opens the door for substrate activation pathways at trinuclear species not available in the former tame-based clusters. Similarly, *C*_3_-symmetric 1,3,5-benzene substituted ligands developed by Holm to mimic iron sulfur clusters,^20,21^ inspired the synthesis of oxygen-donor congeners in work reported by Agapie and coworkers towards the creation of Mn_3_Ca subsite mimics of the oxygen evolving complex.^22–24^ Most recently, Suess *et al.* using a similar ligand base fragment developed a nitrogen-donor analogue capable of isolating an iron sulfur alkyl cluster which mimics elusive enzymatic intermediates.^25,26^ Other systems benefiting from ligand rigidification include those from the Murray group, whereupon limiting the degrees of freedom of their initial cryptand design,^27^ they uncovered [M_3_] clusters, M = Fe, Co, Cu, and Zn, capable of ligating and activating N_2_ and CO_2_.^28–34^ In our case, by increasing the degrees of freedom of our ligand scaffold we have created a binding pocket within an all-Cu(i) [Cu_4_] cluster that binds dioxygen through non-covalent interactions.

## Results and discussion

### Synthetic procedures

**Scheme 1:**
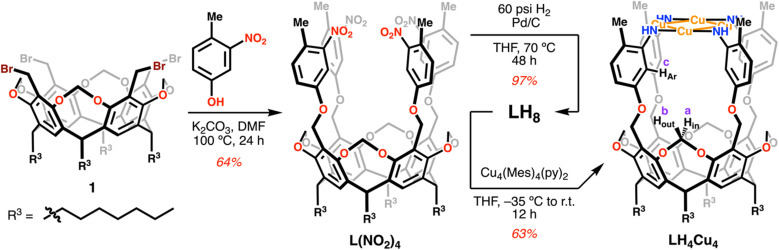
Synthetic pathway for ligand LH_8_ and metalation conditions leading to LH_4_Cu_4_.

Our synthetic protocol provides square planar copper clusters in three steps from 1, a precursor obtained readily in gram-scale quantities,^35^ as shown in Scheme 1. First, a four-fold S_N_2 reaction on 1 by 4-methyl-3-nitrophenol in basic conditions using K_2_CO_3_ in DMF for 24 hours produces ligand precursor L(NO_2_)_4_ in 64% isolated yield. This tetranitro species is reduced under 60 psi of H_2_ over Pd/C in refluxing THF for 48 h. The reaction is quantitative by ^1^H NMR; however, after work up the isolated yield of LH_8_ is 97%. *In situ* deprotonation and metalation of this tetraamine ligand with Cu_4_(Mes)_4_(py)_2_ in THF forms the all-Cu(i) diamagnetic species LH_4_Cu_4_ in 63% yield. ^1^H NMR analysis of LH_4_Cu_4_ reveals its ideal *C*_4v_ symmetry in solution (Fig. S11†).

### Cluster topology

**Fig. 2:**
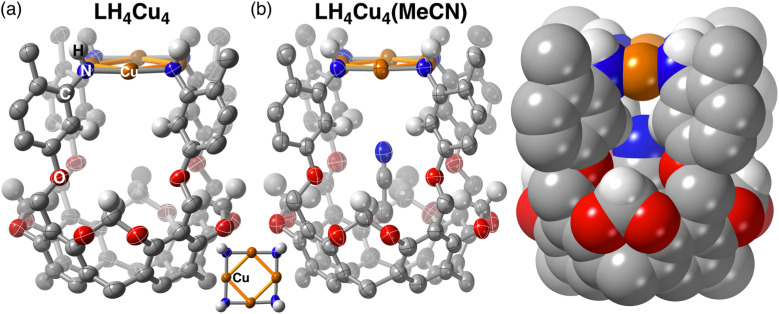
Molecular crystal structure of (a) LH_4_Cu_4_ (100 K) and (b) LH_4_Cu_4_(MeCN) (100 K). Sphere packing model is shown for LH_4_Cu_4_(MeCN) to showcase the congested nature of its internal cavity. The C, N, O, Cu, and H atoms are coloured grey, blue, red, orange, and white, respectively. R groups (*n*-heptyl) and most hydrogen atoms, except for select ones, are omitted for clarity. Thermal ellipsoids are set at 50% probability level.

Single crystal X-ray diffraction data was collected on crystals grown by vapor diffusion of pentane into a concentrated solution of LH_4_Cu_4_ in THF. The molecular structure of LH_4_Cu_4_ confirmed the formation of a square planar arrangement of Cu atoms sitting at an average distance *d*_avg_ (Cu–Cu) of 2.69(2) Å (Fig. 2a). For comparison, in Cu metal the Cu–Cu distance in Cu(100) is 2.55(1) Å.^36^ Additionally, the [Cu_4_] core displays a *d*_avg_ (Cu–N) of 1.89(1) Å and an almost linear local Cu coordination environment with an average ∠N–Cu–N of 177.3(7) degrees. Similar square-shaped tetranuclear copper clusters mimicking Cu_Z_ in N_2_O reductase^37^ have been previously reported by Mankad and coworkers using bridging diphosphine^38,39^ or formamidinate^40–42^ ligands resulting in cluster cores with Cu–Cu distances ranging from around 2.4 to 3.5 Å across all compounds reported therein. In fact, a recent example by the same group demonstrates the formation of a [Cu_4_(O_2_)] adduct, where O_2_ is bound to a single metal site.^43^

LH_4_Cu_4_ is closely related to our previously reported [Cu_4_] clusters;^12,44^ however, we hypothesized that a larger internal cavity should be created in this newly synthesized cluster located in between the [Cu_4_N_4_] fragment and the resorcinarene backbone as a consequence of the axially longer LH_8_ relative to *LH_8_. Our hypothesis was confirmed upon analyzing the molecular crystal structure obtained when LH_4_Cu_4_ is exposed to MeCN (Fig. 2b). The MeCN molecule is hosted below the [Cu_4_] plane establishing a [Cu_4_] centroid-to-N_MeCN_ distance of 3.852 Å. Note that the structure metrics of LH_4_Cu_4_(MeCN) are relatively unchanged (*d*_avg_ (Cu–Cu) = 2.678(5) Å, *d*_avg_ (Cu–N) = 1.890(3) Å, ∠N–Cu–N = 177.4(6) degrees) from LH_4_Cu_4_.

### Host–guest properties

**Fig. 3:**
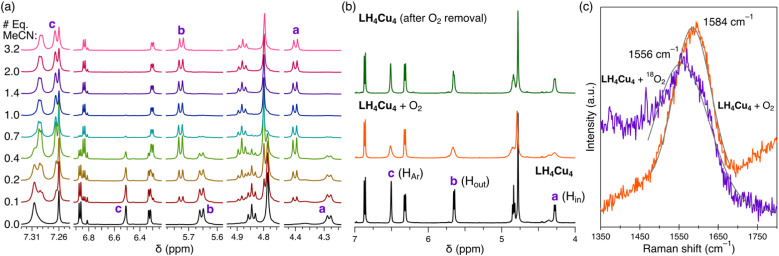
(a) ^1^H NMR spectra indicating resonances a, b, and c as MeCN is titrated into LH_4_Cu_4_ in CDCl_3_ at room temperature under a dinitrogen atmosphere. (b) Sequence of ^1^H NMR spectra of LH_4_Cu_4_ collected under N_2_ (bottom), 1 atm of O_2_ (middle), and last reverting back to N_2_ (top). (c) Variable isotope resonance Raman spectra of LH_4_Cu_4_ plus O_2_ collected at room temperature. The region expected for the O–O stretch is shown. Partial fits (in grey) of the orange and purple traces to Gaussian curves served to locate the maximum.

Titration of MeCN to LH_4_Cu_4_ provided insight into the hydrogen atom resonances involved in the non-covalent bonding of MeCN in the host–guest adduct LH_4_Cu_4_(MeCN). As observed in Fig. 3a, the addition of MeCN in CDCl_3_ leads to resonance shifts in the ^1^H NMR. Particularly noteworthy is that protons a, b, and c, as labeled in Scheme 1, shift downfield as equivalents of MeCN are added (Fig. S15† contains the full spectrum), a well-known effect for H atoms involved in hydrogen bonding.^45,46^ The ^1^H NMR spectrum additionally reveals the location of the MeCN signal at −1.76 ppm. Similar upfield shifts or shielding of the methyl resonance in acetonitrile has been observed in calixarene-based copper clusters.^47^ Moreover, the stepwise addition of MeCN to LH_4_Cu_4_ produces a new set of resonances corresponding to LH_4_Cu_4_(MeCN) while the location of those for LH_4_Cu_4_ remain unchanged, indicating a large host–guest association constant (*K*_a_ > 10^5^ M^−1^ in CDCl_3_) that goes beyond the measurable range *via* NMR.^48^ Variable-temperature ^1^H NMR in C_6_D_6_ provided insight into the thermodynamics of MeCN dissociation from LH_4_Cu_4_(MeCN) (Fig. S16†). A van't Hoff analysis reveals a dissociation enthalpy and entropy of 12.7 kcal mol^−1^ and 25.4 cal mol^−1^ K^−1^ (Fig. S17†), respectively. The calculated binding free energy of −5.14 kcal mol^−1^ at 298 K is similar to solvent binding in cavitands.^49^ Most importantly, structural fidelity of LH_4_Cu_4_(MeCN) in combination with the ^1^H NMR spectroscopic signatures resulting from MeCN binding provides a roadmap to investigate binding of other guests to LH_4_Cu_4_.

Copper–dioxygen chemistry is cornerstone in living systems and has inspired the realization of many synthetic compounds seeking to replicate its structure and function.^50,51^ Seeking to probe potential ligation and activation modes of O_2_ at LH_4_Cu_4_, an all-Cu(i) cluster, we dosed dry O_2_ to an air-free solution of LH_4_Cu_4_ in CDCl_3_. As the atmosphere is exchanged from N_2_ (Fig. 3b bottom) to O_2_ (Fig. 3b middle), we observe broadening in proton resonances a, b, c, and the methine at 4.85 ppm. Note that the same proton resonances a, b, and c, are affected during MeCN binding. These proton resonances sharpen back when the O_2_ atmosphere is removed (Fig. 3b top). Altogether, our experiments indicate that O_2_ is reversibly accommodated in the same binding pocket as MeCN. Note that L(NO_2_)_4_ and LH_8_ do not show any broadening of resonances a, b, and c under the same conditions (Fig. S19 and S20†). It is important to highlight that the vast majority of synthetic molecular [Cu^I^_*n*_] systems, with nuclearities ranging from *n* = 1 to 4, engage O_2_ by establishing a copper–oxygen covalent bond giving rise to terminal or bridging peroxo, superoxo, or oxo complexes.^52^ To the best of our knowledge, this is the first formally all-Cu(i) cluster reversibly binding O_2_ solely *via* non-covalent interactions.

Further examination of O_2_ binding to LH_4_Cu_4_ was carried out by collecting resonance Raman vibrational spectra on dried thin films of LH_4_Cu_4_ contained within a quartz cuvette either under vacuum or dry dioxygen atmosphere. Laser excitation at 532 nm gave a band centered at 1584 cm^−1^ assigned to the O–O stretching of dioxygen hosted within LH_4_Cu_4_ (Fig. 3c). Note that the symmetrical stretching band in free O_2_ appears at 1556.4 cm^−1^ (Fig. S22†).^53^ The same set of experiments were executed using isotopically labeled ^18^O_2_ displaying a stretching band at 1556 cm^−1^. The simple harmonic oscillator model predicts a shift of ∼90 cm^−1^, however we only observe a Δ^18^O of 28 cm^−1^.^52^ We hypothesize that dioxygen enters the cavity of LH_4_Cu_4_ likely through partial ligand dissociation or *via* the formation of a dilated aperture.^54,55^ Altogether, it appears that the non-covalent interactions serve to stabilize the O_2_ molecule within LH_4_Cu_4_ and at the same time strengthens the O–O bond by removing π* electron density.

To further investigate the electronic properties of LH_4_Cu_4_ and its adduct with O_2_, we employed density functional theory (DFT) methods. 
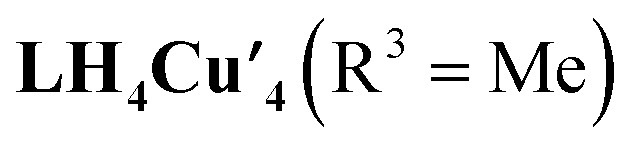
 was optimized at B3LYP-D3BJ/Def2-SVP+PCM(CHCl_3_) level of theory. The optimized structure reproduced the experimental data with high accuracy as determined by overlaying these structures and obtaining a root mean square displacement (RMSD) of 0.3 Å and *d*_avg_ (Cu–Cu) of 2.67 Å (Fig. S23†), indicating the reliability of the selected method.

**Fig. 4:**
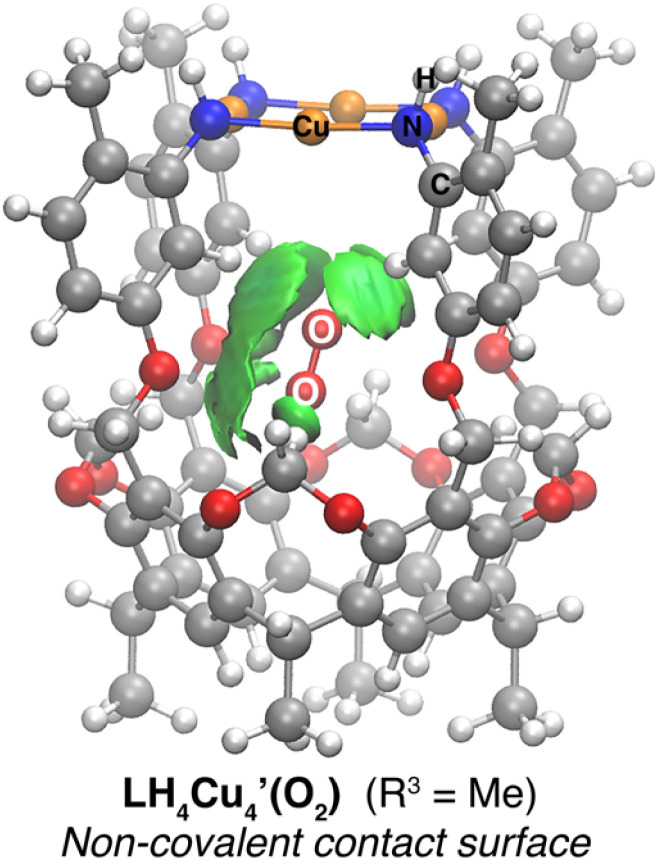
IGMH map displaying the non-covalent interactions between O_2_ and LH_4_Cu_4_ (isovalue = 0.0011 a.u.).

Intrigued by the non-covalent binding of O_2_ to LH_4_Cu_4_, the adduct 
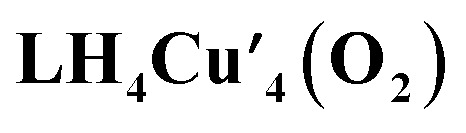
 was first optimized employing B3LYP-D3BJ/Def2-SVP+PCM(CHCl_3_) level of theory. The binding pocket within 
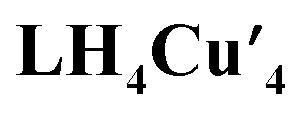
 is best visualized by applying the independent gradient model based on Hirshfeld partition of molecular density (IGMH),^56^ which clearly reveals the contact surface of weak non-covalent interactions involved in hosting the O_2_ molecule. Note that the oxygen molecule positions itself in a way to maximize interactions with the C–H bonds from the bridging methylenes and top aromatic rings, protons a and c, respectively, in Scheme 1. The O_2_ molecule was placed at different starting positions during structure optimization, *e.g.*, close to the resorcin[4]arene base, near the Cu_4_ plane, and in all cases the optimum location found is that shown in Fig. 4. Overall, the IGMH isosurface for the O_2_ adduct is well supported experimentally as reflected in the ^1^H NMR data shown in Fig. 3b.

## Conclusions

Here, we designed a novel ligand architecture carrying four aniline moieties to obtain a rigidified square-planar [Cu_4_] topology despite the ligand's rotational degrees of freedom. The foregoing results show that, changing the resorcinarene backbone employed previously by our group engenders a cluster compound with a cavity to encapsulate small molecules, where we showcase a unique example of reversible non-covalent binding of O_2_ within the [Cu_4_] cluster built around a supramolecular scaffold.

## Data availability

All data including experimental and analytical details are in the ESI.†

## Author contributions

Manasseh K. Osei: conceptualization, investigation, data curation, writing – review & edition. Saber Mirzaei: conceptualization, investigation, data curation, formal analysis, writing – review & edition. M. Saeed Mirzaei: investigation, data curation, formal analysis. Agustin Valles: investigation. Raúl Hernández Sánchez: conceptualization, project administration, resources, funding acquisition, supervision, visualization, writing – original draft, writing – review & edition.

## Conflicts of interest

There are no conflicts to declare.

## Supplementary Material

SC-015-D3SC06390A-s001

SC-015-D3SC06390A-s002
