# Supplementary material for: Reversible dioxygen uptake at [Cu4] clusters
Source: Chem Sci. 2024 Mar 7;15(14):5327–32. doi: 10.1039/d3sc06390a (PMC10988628; doi:10.1039/d3sc06390a)
Supplement: SC-015-D3SC06390A-s001 [file SC-015-D3SC06390A-s001.pdf]

Electronic Supplementary Material (ESI) for Chemical Science.
This journal is © The Royal Society of Chemistry 2024

Supplementary Information for:

"Reversible dioxygen uptake at [Cu_4] clusters"

Manasseh Kusi Osei,[1] Saber Mirzaei,[1,2] M. Saeed Mirzaei,[1] Agustin Valles,[1] and Raúl Hernández Sánchez[1,2]*

[1]Department of Chemistry, Rice University,
6100 Main St., Houston, Texas, 77005, USA.
[2]Department of Chemistry, University of Pittsburgh,
219 Parkman Ave., Pittsburgh, Pennsylvania, 15260, USA.

Corresponding author e-mail: raulhs@rice.edu

Table of Contents

Experimental section S4

Table S1: Crystallographic data for compounds $\mathbf{LH_4Cu_4}$ and $\mathbf{LH_4Cu_4(MeCN)}$. S12

Figure S1. Molecular crystal structure of $\mathbf{LH_4Cu_4}$. S13

Figure S2. Molecular crystal structure of $\mathbf{LH_4Cu_4(MeCN)}$. S14

Figure S3. ^{1}H and ^{13}C NMR spectra of compound **S2**. S15

Figure S4. ^{1}H and ^{13}C NMR spectra of compound **S1**. S16

Figure S5. ^{1}H and ^{13}C NMR spectra of compound **1**. S17

Figure S6. COSY NMR spectrum of compound **1**. S18

Figure S7. ^{1}H and ^{13}C NMR spectra of $\mathbf{L(NO_2)_4}$. S19

Figure S8. COSY NMR spectrum of $\mathbf{L(NO_2)_4}$. S20

Figure S9. ^{1}H and ^{13}C NMR spectra of compound $\mathbf{LH_8}$. S21

Figure S10. COSY NMR spectrum of compound $\mathbf{LH_8}$. S22

Figure S11. ^{1}H and ^{13}C NMR spectra of $\mathbf{LH_4Cu_4}$. S23

Figure S12. COSY NMR spectrum of $\mathbf{LH_4Cu_4}$. S24

Figure S13. Cyclic voltammetry of $\mathbf{LH_4Cu_4}$ in THF. S25

Figure S14. ^{1}H NMR spectrum of $\mathbf{LH_4Cu_4(MeCN)}$. S26

Figure S15. ^{1}H NMR spectra of MeCN titration into $\mathbf{LH_4Cu_4}$. S27

Figure S16. VT ^{1}H NMR spectra of MeCN dissociation from $\mathbf{LH_4Cu_4(MeCN)}$. S28

Figure S17. Van't Hoff analysis of MeCN dissociation from $\mathbf{LH_4Cu_4(MeCN)}$. S29

Figure S18. ^{1}H NMR spectra of $\mathbf{LH_4Cu_4}$ exposed to N_2 and O_2 atmospheres. S30

Figure S19. ^{1}H NMR spectra of $\mathbf{L(NO_2)_4}$ exposed to N_2 and O_2 atmospheres. S31

Figure S20. ^{1}H NMR spectra of $\mathbf{LH_8}$ exposed to N_2 and O_2 atmospheres. S32

Table S2. Diffusion coefficients extracted from DOSY NMR for $\mathbf{LH_4Cu_4}$. S33

Figure S21. UV-vis and emission spectra of $\mathbf{LH_4Cu_4}$ exposed to N_2 and O_2. S34

Figure S22. Resonance Raman spectra of $\mathbf{LH_4Cu_4}$ plus O_2. S35

Figure S23. RMSD between $\mathbf{LH_4Cu_4}$ experimental and its DFT-calculated structure. S36

Table S3. DFT calculated ^{1}H NMR data for $\mathbf{LH_4Cu_4'}$. S37

Cartesian coordinates of DFT optimized $\mathbf{LH_4Cu_4'}$ and $\mathbf{LH_4Cu_4'(O_2)}$. S38

References S46

Experimental section

General considerations. All manipulations involving metal complexes were performed under an atmosphere of dry, oxygen-free N_2 by means of standard Schlenk or glovebox techniques (MBraun glovebox equipped with a –35 ºC freezer). All glassware was oven dried for a minimum of 10 h and cooled in an evacuated antechamber prior to use in the drybox. Anhydrous and anaerobic tetrahydrofuran (THF), diethyl ether (Et_2O), and hexanes were dried and deoxygenated on dual high-performance columns within a Glass Contour 800L Solvent Purification System and stored over 4 Å molecular sieves prior to use. Chemicals and solvents were purchased from commercial suppliers and used as received. Compound **1** has been reported previously,[1] however it was synthesized through a modified procedure akin to previous reports.[2]

^1H- and ^{13}C- nuclear magnetic resonance (NMR) spectra were obtained on any of the following instruments: a) Bruker Avance 400 spectrometer at 400 and 100 MHz, b) Bruker Avance 500 spectrometer at 500 and 125 MHz, c) Bruker Avance 600 spectrometer at 600 and 150 MHz, respectively. Chemical shifts for protons are reported in parts per million (ppm) downfield from tetramethylsilane (TMS) and are referenced to residual protium in the NMR solvent ($CHCl_3$: δ 7.26). Chemical shifts for carbon are reported in ppm downfield from TMS and are referenced to the carbon resonances of the solvent ($CDCl_3$: δ 77.0;). Data are presented as follows: chemical shift, multiplicity (s = singlet, d = doublet, t = triplet, m = multiplet, br = broad), coupling constants in Hertz, and integration.

High-resolution mass spectrometry (HRMS) was performed on a (1) Thermo Scientific Q-Exactive Oribtrap instrument equipped with a Dionex Ultimate 3000 (RSLC) inlet system, and electrospray (ESI) and atmospheric pressure chemical (APCI) ionization sources.

Resonance Raman spectra were collected on a Renishaw Invia Raman Microscope. The usual experiment consisted of dissolving **LH_4Cu_4** in THF which was drop-cast into a quartz cuvette to create a thin film. The quartz cuvette was modified with a J Young valve in order to introduce gaseous samples as desired. Data was collected using a 532 nm laser at 5% power, 10 s acquisition time, and 2 acquisitions. Microscope zoom was 50x.

Absorption spectra were obtained on a Cary 60 UV-Vis spectrophotometer and emission spectra were recorded in a HORIBA Jobin Yvon Fluorolog-3 spectrofluorometer.

Single crystal data for **LH_4Cu_4** and **LH_4Cu_4(MeCN)** were collected on a Rigaku Synergy-S diffractometer equipped with dual-beam microfocus Cu and Mo radiation sources paired with a Rigaku's HyPix-Arc150 detector. Temperature was maintained using an Oxford Cryosystem nitrogen flow apparatus. Single crystals of **LH_4Cu_4** and **LH_4Cu_4(MeCN)** suitable for X-ray structure analysis were coated with Paratone N-oil and mounted on MiTeGen Kapton loops (polyimide). Data collected was integrated and corrected using CrysAlisPro V42. Space group assignments were determined by examination of systematic absences, E-statistics, and successive refinement of the structures. The program PLATON[3-4] was employed to confirm the absence of higher symmetry for any of the crystals. The positions of the heavy atoms were determined using intrinsic phasing methods using the program SHELXT[5] and SHELXL[6] with Olex2[7] interface. Successive cycles of least-square refinement followed by difference Fourier syntheses revealed the positions of the remaining non-hydrogen atoms. Non-hydrogen atoms were refined with anisotropic displacement parameters, and hydrogen atoms were added in idealized positions. Crystallographic data for **LH_4Cu_4** and **LH_4Cu_4(MeCN)** are given in Table S1. Error on the average distances was calculated according to published procedures.[8]

Synthetic details. The synthesis of resorcin[n]arenes is well documented.[9] However, to the best of our knowledge, compound **S2** has not been reported before since compound **1** was obtained through a different route.[1-2] Thus, we include its synthetic protocol and characterization. **$Cu_4(Mes)_4(py)_2$** was prepared as recently described in the literature.[10] Compound **S1** was prepared according to published procedures.[2]

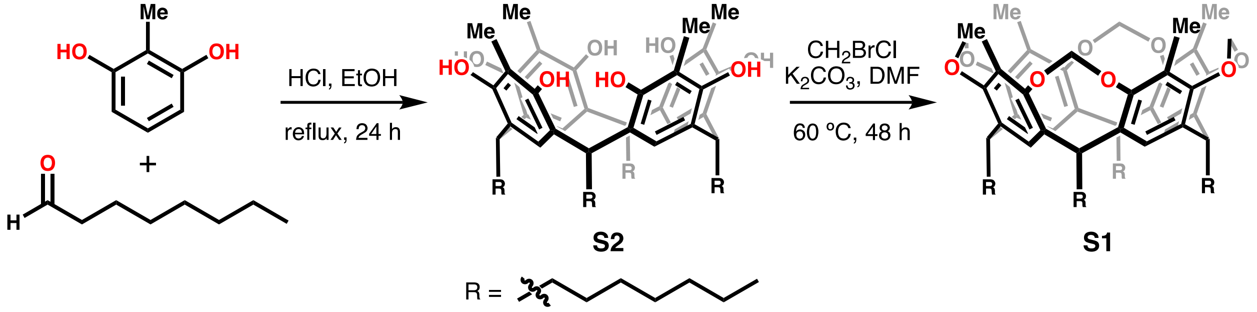

Compound **S2**. To a 1 L Schlenk flask containing 2-methylresorcinol (30 g, 241.6 mmol) was added octanal (31 g, 241.6 mmol) in EtOH (400 mL). 100 mL HCl was slowly added to the Schlenk flask and the mixture left to stir vigorously for 1 h at room temperature. Subsequently it was brought to reflux for 24 h. The reaction mixture was cooled down to room temperature after which ~400 mL water was added to the flask. A precipitate was formed which was filtered and washed with water to obtain the product as a yellow-orange solid. Yield: 90% (51 g, 217.4 mmol)

^{1}H NMR (400 MHz, $(CD_3)_2CO$, 20 ºC): δ 7.94 (s, 8H), 7.40 (s, J = 7.8 Hz, 4H), 4.38 (t, J = 7.8 Hz, 4H), 2.28 (q, J = 14.5, 7.7 Hz, 8H), 2.04 (s, J = 3.4 Hz, 12H), 1.29 (m, 40H), and 0.89 (d, J = 6.7 Hz, 12H) ppm.

^{13}C NMR (100 MHz, $(CD_3)_2CO$, 20 ºC): δ 150.4, 125.6, 122.1, 112.0, 35.4, 34.8, 32.7, 30.5, 29.1, 23.3, 14.4, and 9.8 ppm. $C_{60}H_{89}O_8$, HRMS $[M+H]^+$ calc.:937.6552; exp.: 937.6521.

Compound **S1**. A 500 mL three-neck flask was loaded with **S2** (20 g, 21.3 mmol), K_2CO_3 (23.5 g, 170.4 mmol) in 300 mL DMF. The mixture was put under nitrogen bubbling for 3 hours after which 7 mL of CH_2BrCl was added through the rubber septum of one neck (the central neck was equipped with a condenser and connected to an oil bubbler). The reaction was stirred at 60 ºC for 48 h. During this time, 3 more portions of 7 mL CH_2BrCl were added at 12 h intervals. The DMF was removed after 48 hours and the solid obtained was washed with water. The crude product is

purified by vacuum chromatography using 50-100% DCM in hexanes. The product was obtained as an off-white solid. Yield: 43% (9.0 g, 9.2 mmol).

^{1}H NMR (400 MHz, $CDCl_3$, 20 ºC): δ 6.99 (s, 4H), 5.89 (d, *J* = 6.9 Hz, 4H), 4.77 (t, *J* = 8.1 Hz, 4H), 4.27 (d, *J* = 6.9 Hz, 4H), 2.22 (q, *J* = 14.8, 7.9 Hz, 8H), 1.98 (s, 12H), 1.30 (m, 40H), and 0.89 (d, *J* = 6.8 Hz, 12H) ppm.

^{13}C NMR (100 MHz, $CDCl_3$, 20 ºC): δ 153.4, 138.1, 123.7, 117.7, 98.6, 37.1, 32.0, 30.3, 29.5, 28.1,22.8, 14.3, and 10.5 ppm. $C_{64}H_{89}O_8$, HRMS $[M+H]^+$ calc.:985.6552; exp.: 985.6524.

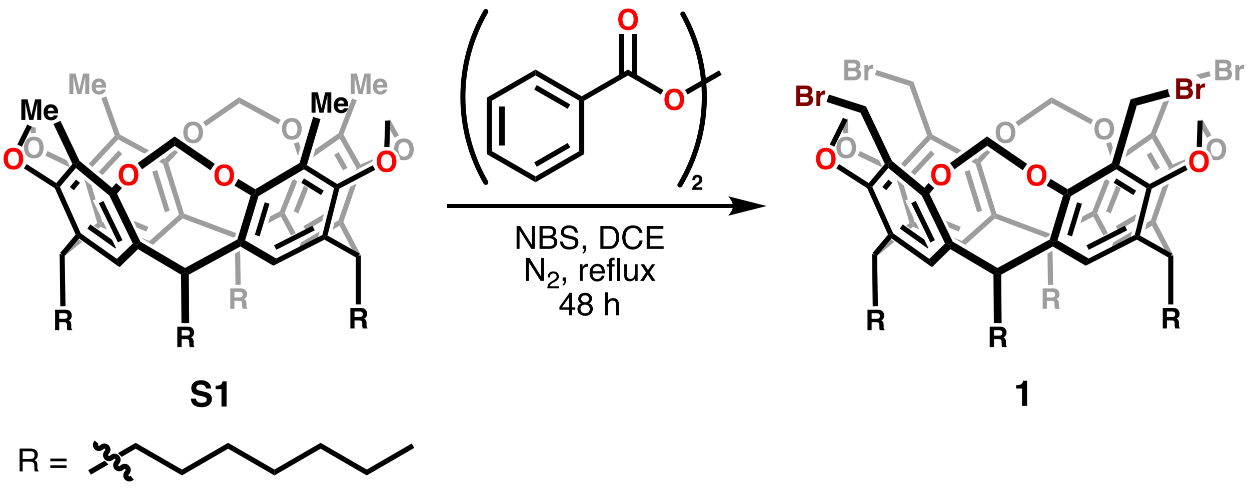

S1 **1**

Compound **1**. To a 250 mL Schlenk flask was added **S1** (4.5 g, 4.6 mmol) and dissolved in 200 mL of DCE. The mixture was degassed for 20 min. Subsequently, NBS (4.1 g, 23 mmol) and benzoyl peroxide (0.3 g) were added. The reaction mixture was degassed for an additional 15 min and later heated to reflux under N_2 atmosphere for 1 h. Then another 0.7 g of benzoyl peroxide was added while keeping the flask under nitrogen flow. After 48 h of reflux, the solvent was removed under vacuum. The solid was transferred and passed through a flash column using pure DCM to obtain a light yellow solid. Yield: 77% (4.6 g, 3.5 mmol).

^{1}H NMR (400 MHz, $CDCl_3$, 20 ºC): δ 7.13 (s, 4H), 6.02 (d, *J* = 6.6 Hz, 4H), 4.78 (t, *J* = 8.0 Hz, 4H), 4.55 (d, *J* = 6.5 Hz, 4H), 4.42 (s, 8H), 2.20 (q, *J* = 14.6, 7.8 Hz, 8H), 1.29 (m, 40H), and 0.89 (d, *J* = 6.8 Hz, 12H) ppm.

^{13}C NMR (100 MHz, $CDCl_3$, 20 ºC): δ 153.7, 138.2, 124.6, 121.1, 99.2, 37.0, 32.0, 30.2, 29.9, 29.5, 28.0, 23.1, 22.8, and 14.2 ppm. $C_{92}H_{108}N_4O_{20}$, HRMS could not be obtained successfully for this compound.

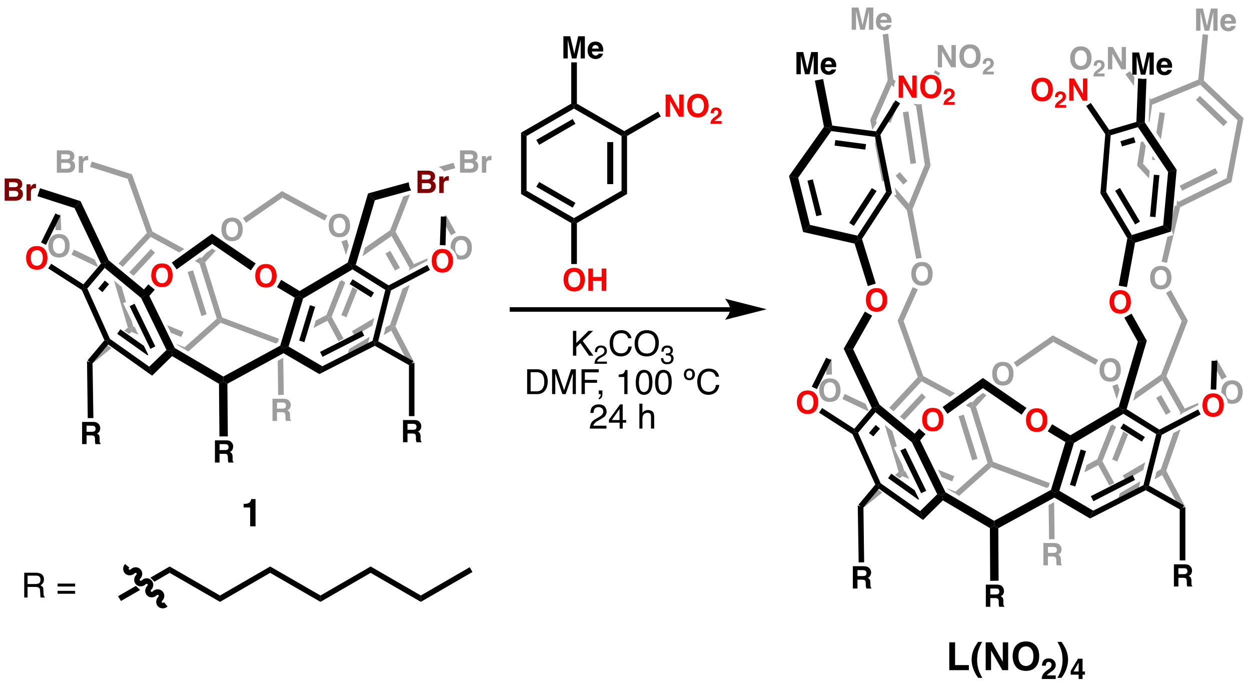

Compound **L(NO₂)₄**. To a 250 mL round flask containing **1** (2.5 g, 1.9 mmol), K_2CO_3 (2.66 g, 10 eq) and 4-methyl-3-nitrophenol (1.48 g, 9.66 mmol) was added 100 mL of DMF. The mixture was stirred for 24 h at 100 ºC. The resulting mixture was cooled to room temperature and the solvent was removed under vacuum. The resulting solid was suspended in water and filtered using filter paper. The residue was washed with more water to remove K_2CO_3.The crude product was further purified by column chromatography using DCM/hexanes (1/1, v/v) giving the product as a white solid. Yield: 64% (1.97 g, 1.2 mmol).

^1H NMR (400 MHz, $CDCl_3$, 20 ºC): δ 7.54 (d, J = 2.5 Hz, 4H), 7.28 (s, J = 8.0 Hz 4H), 7.22 (d, J = 8.5 Hz, 4H), 7.03 (dd, 4H), 5.75 (d, J = 7.2 Hz, 4H), 4.93 (s, 8H), 4.85 (t, J = 8.0 Hz, 4H), 4.59 (d, J = 7.2 Hz, 4H), 2.51 (s, 12H), 2.29 (m, 8H), 1.39 (m, 40H), and 0.91 (t, J 6.6 = Hz, 12H) ppm.

^{13}C NMR (100 MHz, $CDCl_3$, 20 ºC): δ 157.1, 154.5, 149.6, 138.3, 133.9, 126.3, 122.2, 121.7, 120.3, 109.9, 100.1, 61.4, 37.0, 32.0, 30.3, 29.9, 29.5, 28.0, 22.8, and 14.3 ppm. $C_{92}H_{108}O_{20}N_4$, HRMS could not be obtained successfully for this compound.

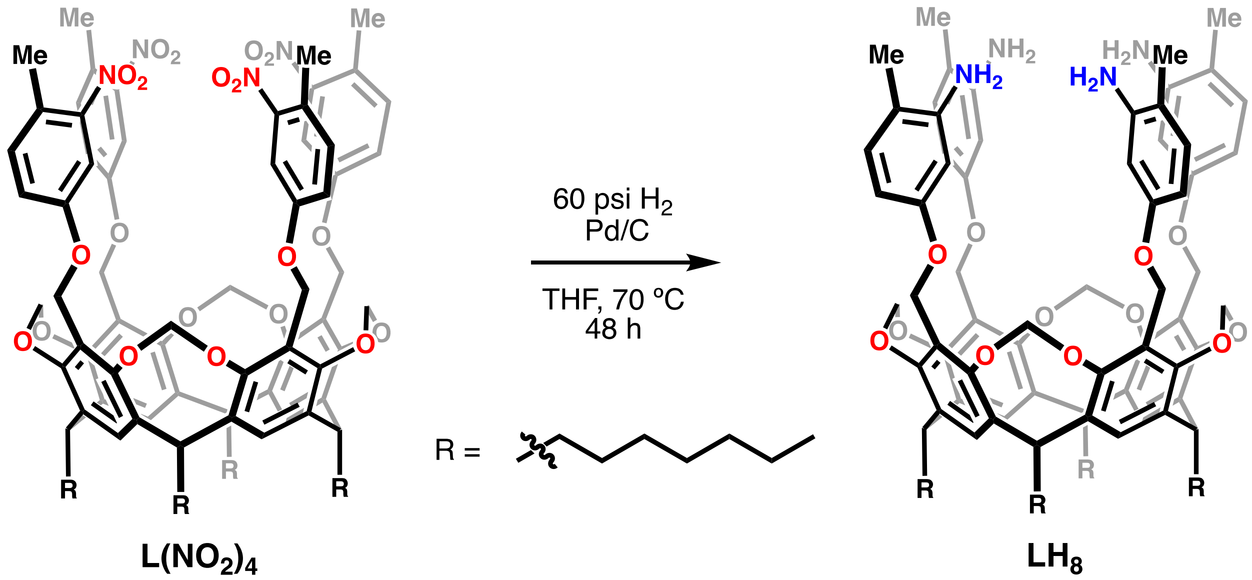

Compound **LH_8**. **$L(NO_2)_4$** (1.0 g, 0.63 mmol), 10% Pd/C (100 mg) and anhydrous THF (70 mL) were all charged into a Parr reactor and stirred under 60 psi of H_2 at 70 ºC. The completion of the reaction was confirmed by thin layer chromatography at 48 hours. The reaction mixture was filtered through a plug of celite on a fritted glass funnel and washed with anhydrous THF. The THF was removed under vacuum to give the product as an off white solid. Yield: 97% (0.90 g, 0.61 mmol).

^{1}H NMR (400 MHz, $CDCl_3$, 20 ºC): δ 7.23 (s, 4H), 6.91 (d, J = 8.3 Hz 4H), 6.31 (dd, J = 8.2 Hz, 4H), 6.20 (d, J = 2.0 Hz 4H), 5.74 (d, J = 7.3 Hz, 4H), 4.85 (d, J = 10.6 Hz, 12H), 4.66 (d, J = 7.4 Hz, 4H), 3.54 (s, 4H), 2.26 (d, 8H), 2.08 (s, 12H), 1.37 (m, 40H), and 0.91 (t, J = 6.1 Hz, 12H) ppm.

^{13}C NMR (100 MHz, $CDCl_3$, 20 ºC): 158.5, 154.6, 145.6, 138.0, 131.1, 123.3, 121.0, 115.3 104.2, 102.2, 100.4, 60.8, 37.0, 32.0, 30.4, 30.0, 29.5, 28.1, 22.8, 16.7 and 14.3 δ ppm. $C_{92}H_{116}N_4O_{12}$, HRMS $[M+H]^+$ calc.: 1469.8663; exp.: 1469.8662.

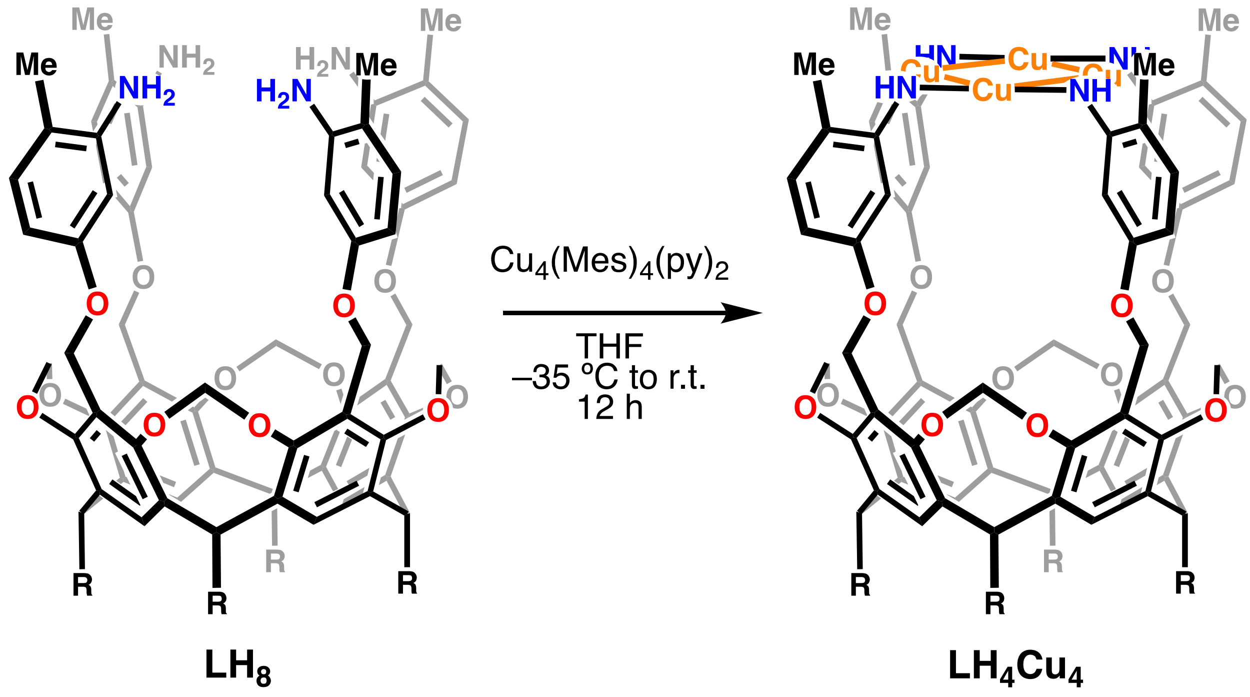

Cluster **LH_4Cu_4**. THF (10 mL) was added to **LH_8** (0.1 g, 0.068 mmol) in a 20 mL scintillation vial and the solution was placed in the freezer at –35 ºC. After 15 minutes, a pre-cooled solution of $Cu_4(Mes)_4(py)_2$ (0.062 g, 0.069 mmol) in THF at –35 ºC was added dropwise to the **LH_8** solution and stirred at room temperature for 12 hours. All volatiles were removed from the off-white solution and pentane was added to form a suspension. The pentane suspension was filtered over celite and washed with more pentane (2 × 5 mL) and hexanes (2 × 5 mL). The residual solid on celite was washed into a separate vial with THF and dried to get an off-white solid. The resulting solid was further washed with diethyl ether (2 × 5 mL). The remaining solid was dried under vacuum and redissolved in THF. This solution was filtered through a plug of celite to afford **LH_4Cu_4**. Yield: 63% (0.074 g, 0.043 mmol). High quality crystals were grown via diffusion of pentane into a concentrated solution of **LH_4Cu_4** in THF. **LH_4Cu_4** was washed with MeCN and high-quality crystals of **LH_4Cu_4(MeCN)** were grown by diffusing pentane into a THF solution of the MeCN-washed product.

^{1}H NMR (500 MHz, $CDCl_3$, 20 ºC): δ 7.30 (s, 4H), 6.86 (d, *J* = 8.2 Hz 4H), 6.50 (s, 4H), 6.32 (d, 4H), 5.65 (d, *J* = 7.4 Hz 4H), 4.84 (t, *J* = 8.1 Hz 4H), 4.78 (s, 8H), 4.28 (d, *J* = 7.2 Hz 4H), 2.63 (s, 4H), 2.30 (q, *J* = 7.9 Hz 8H), 2.07 (s, 12H), 1.40 (m, 40H), and 0.90 (t, *J* = 6.7 Hz 12H).

^{13}C NMR (151 MHz, $CDCl_3$, 20 ºC): δ 158.5, 154.6, 152.0,137.8, 130.9, 127.1, 123.7, 121.0, 117.9, 109.0, 101.6, 101.1, 59.5, 37.0, 32.0, 30.2, 30.0, 29.6, 28.1, 22.8, 22.5, 21.4, 17.2, and 14.3 ppm. $C_{92}Cu_4H_{112}N_4O_{12}$, HRMS was attempted multiple times, but it could not be obtained.

Computational details. All calculations were carried out using Gaussian 16 software package.[11] B3LYP[12] functional of density functional theory (DFT) is used for all optimizations and subsequent studies of neutral molecules. The frequency calculations were carried out for all optimized structures to ensure the absence of any imaginary frequencies for the ground state molecules. In order to include the dispersion effects, the D3 version of Grimme with Becke-Johnson damping factors (D3BJ) were used.[13] The double-zeta quality basis sets (Def2-SVP) of Ahlrichs def2 basis set family were used for all calculations.[14] The implicit solvation effects were included using the integral equation formalism variant of the polarizable continuum model (IEF-PCM) with standard parameters of $CHCl_3$.[15] The gauge-independent atomic orbital (GIAO) method[16] was used for ^1H NMR chemical shift calculations. The calculated chemical shieldings were scaled with ^1H NMR chemical shift of TMS calculated at the same level of theory. The DFT calculated ^1H NMR chemical shifts assisted in assigning the experimental spectra. In order to decrease the computational costs, the C_7 tail of the resorcin[4]arene was replaced with methyl groups. The optimized geometry of **$LH_4Cu_4'(O_2)$** was further used to generate the wavefunction file for the visualization of weak interactions based on the independent gradient model using Hirshfeld partition of molecular density (IGMH) method.[17] The isosurface map was rendered by VMD 1.9.3 program[18] based on the cube files generated by MultiWFN 3.8.[19]

Table S1. Crystallographic data for compounds **LH_4Cu_4** and **LH_4Cu_4(MeCN)**.

	**LH_4Cu_4**	**LH_4Cu_4(MeCN)**
**CCDC Number**	2332560	2333831
**Chemical formula**	$C_{96}H_{120}Cu_4N_4O_{13}$ $\cdot C_5H_{12}$	$C_{92}H_{110}Cu_4N_4O_{12}$ $\cdot C_2H_3N\cdot 0.25(C_4H_8O)$
**Formula weight**	1864.26	1765.06
**Space group**	*R–3*	*P–1*
a **(Å)**	51.7208(3)	14.3434(3)
b **(Å)**	51.7208(3)	18.4638(5)
c **(Å)**	23.7243(2)	19.1152(5)
α **(deg)**	90	107.861(2)
β **(deg)**	90	104.915(2)
γ **(deg)**	120	94.308(2)
V **($Å^3$)**	54961.0(8)	4590.2(2)
Z	18	2
**μ(mm^{-1})**	1.17	1.53
**T (K)**	100	100
***R*1[a] (*wR*2[b])**	0.101 (0.341)	0.105 (0.351)
**Reflections**	23802	17927
**Radiation type**	Cu $K\alpha$	Cu $K\alpha$

[a]$R1 = [\Sigma w(F_o - F_c)^2/\Sigma wF_o^2]^{1/2}$; [b]$wR2 = [\Sigma[w(F_o^2 - F_c^2)^2]/\Sigma w(F_o^2)^2]^{1/2}]$, $w = 1/[\sigma^2(F_o^2) + (aP)^2 + bP]$, where $P = [\max(F_o^2,0) + 2(F_c^2)]/3$

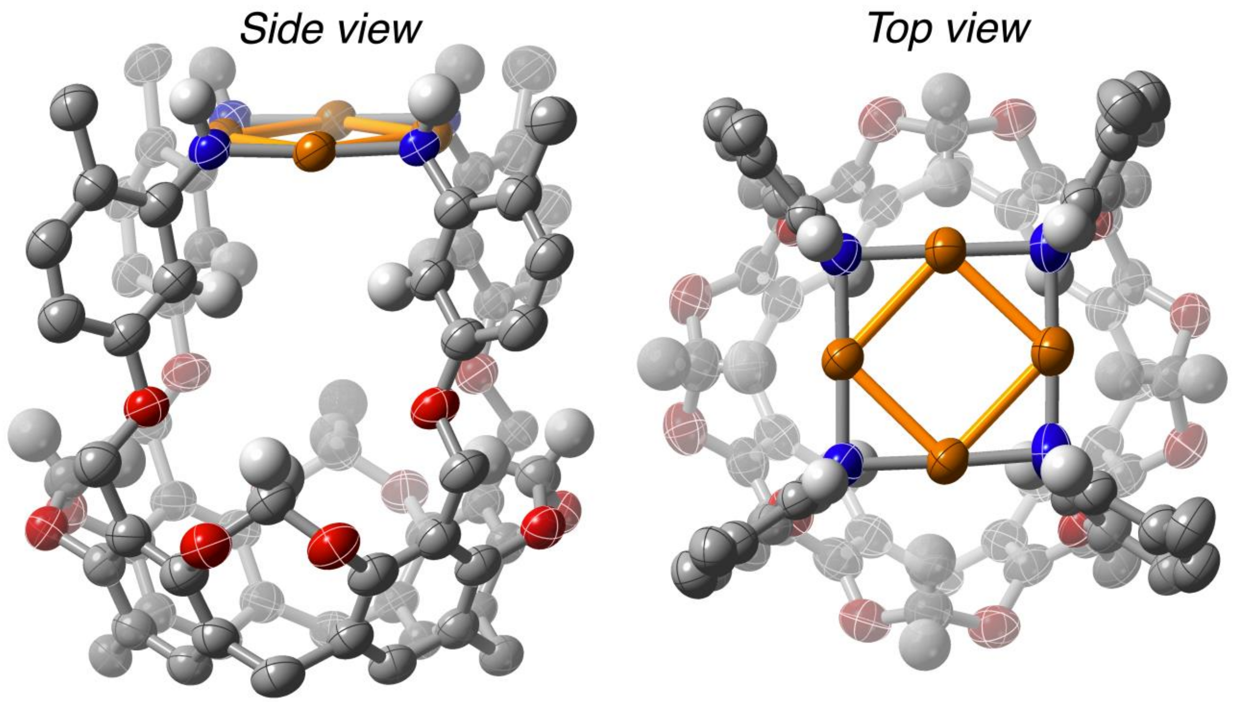

Figure S1. Molecular crystal structure of **LH_4Cu_4** obtained at 100 K. Thermal ellipsoids are set at 50% probability level. Hydrogen atoms are omitted for clarity. The C, Cu, N, O, and H atoms are colored grey, orange, blue, red, and white, respectively. For **LH_4Cu_4**, d_{avg} (Cu–Cu) = 2.69(2) Å, d_{avg} (Cu–N) = 1.89(1) Å, and ∠N–Cu–N = 177.3(7) degrees.

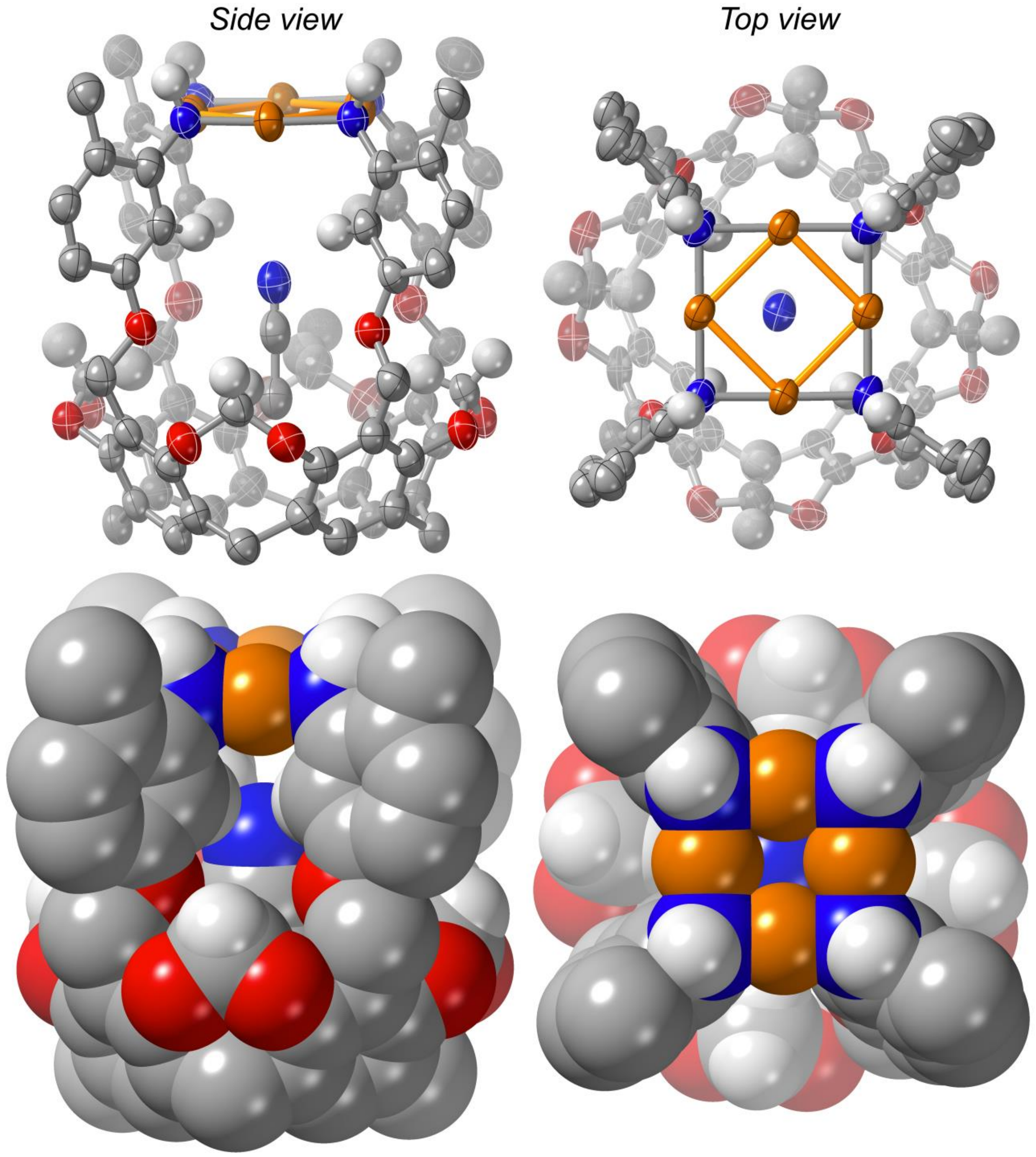

Figure S2. Molecular crystal structure of **LH_4Cu_4(MeCN)** obtained at 100 K. Thermal ellipsoids are set at 50% probability level. Hydrogen atoms are omitted for clarity. The C, Cu, N, O, and H atoms are colored grey, orange, blue, red, and white, respectively. For **$^{Me}LH_4Cu_4$(MeCN)**, d_{avg} (Cu–Cu) = 2.678(5) Å, d_{avg} (Cu–N) = 1.890(3) Å, ∠N–Cu–N = 177.4(6) degrees, d_{avg} ($Cu_{4\ centroid}$–N_{MeCN}) = 3.852 Å, and d_{avg} ($Aryl_{centroid}$–$C_{\mathit{Me}CN}$) = 3.72(1) Å.

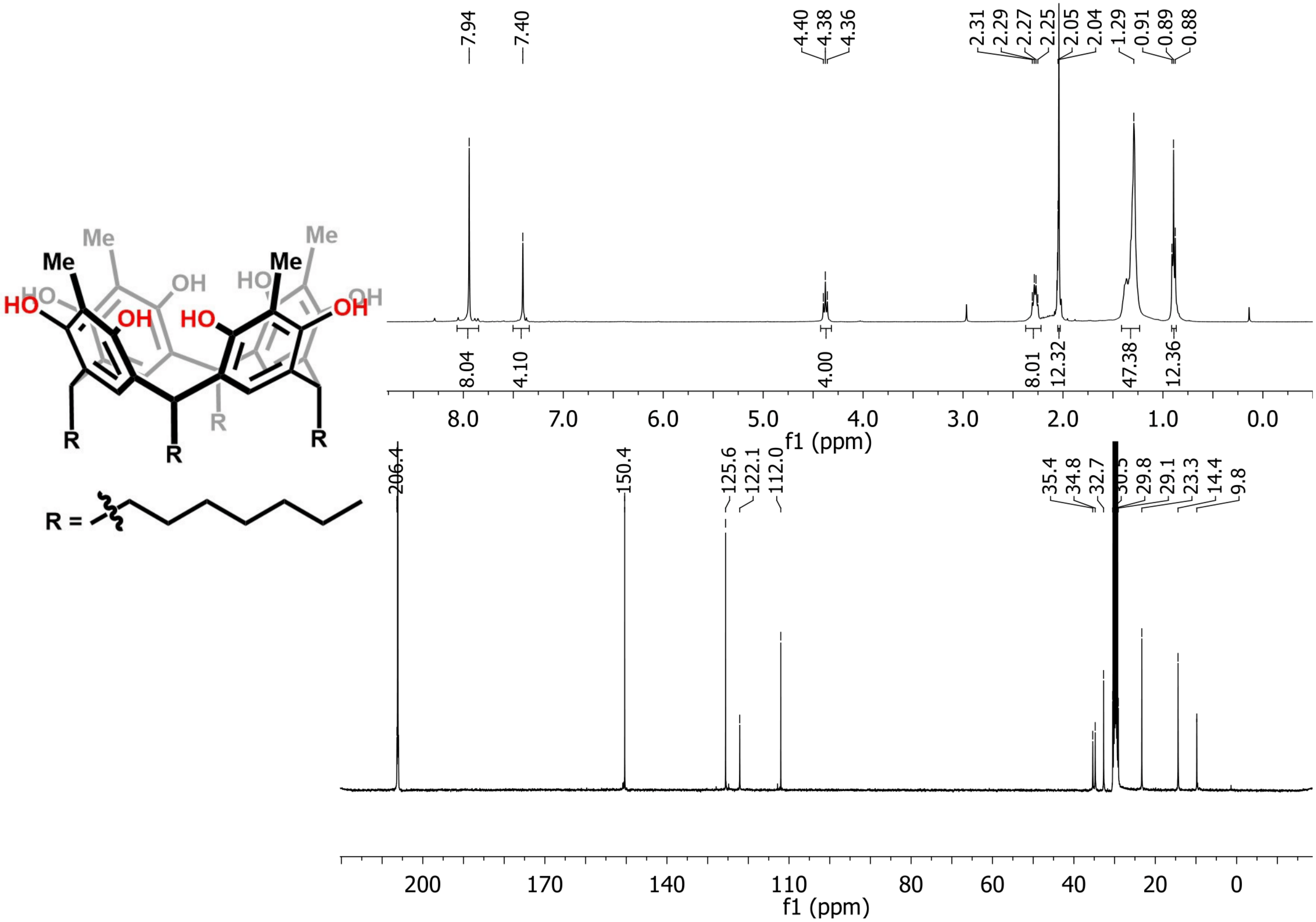

Figure S3. ^1H and ^{13}C NMR spectra of **S2**. Data collected in $(CD_3)_2CO$ at 20 ºC.

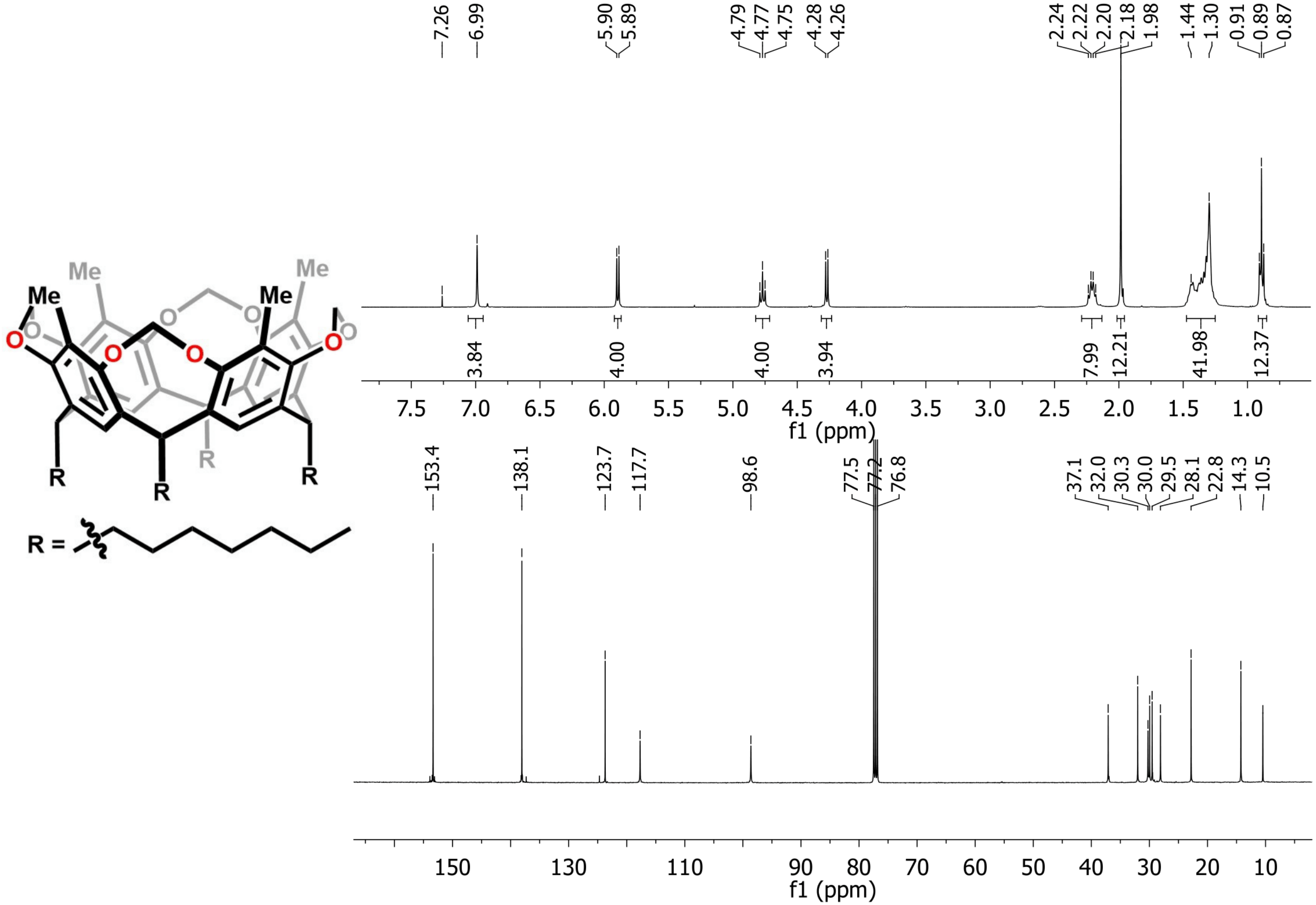

Figure S4. ^{1}H and ^{13}C NMR spectra of **S1**. Data collected in $CDCl_3$ at 20 ºC.

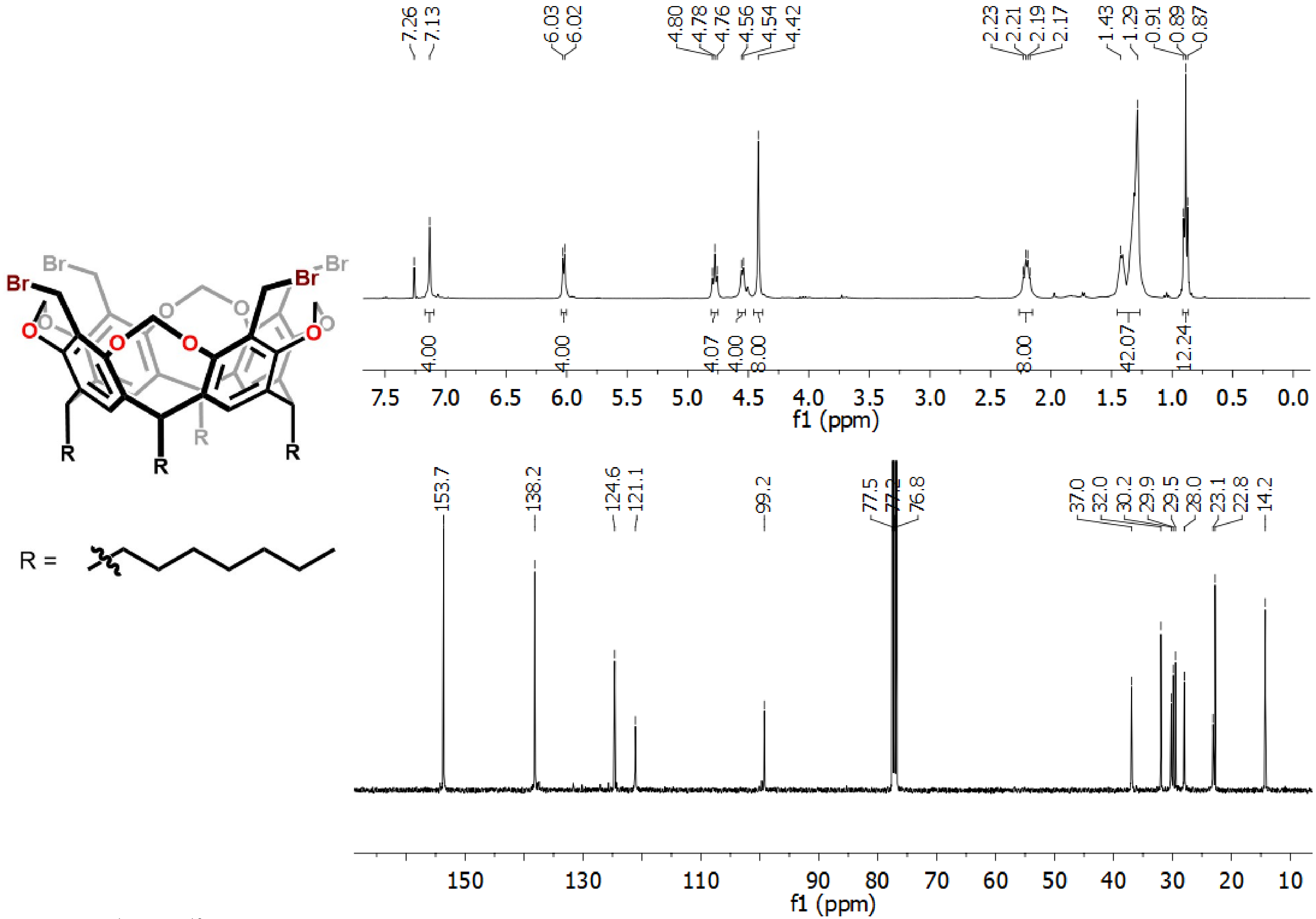

Figure S5. ^{1}H and ^{13}C NMR spectra of **1**. Data collected in $CDCl_3$ at 20 ºC.

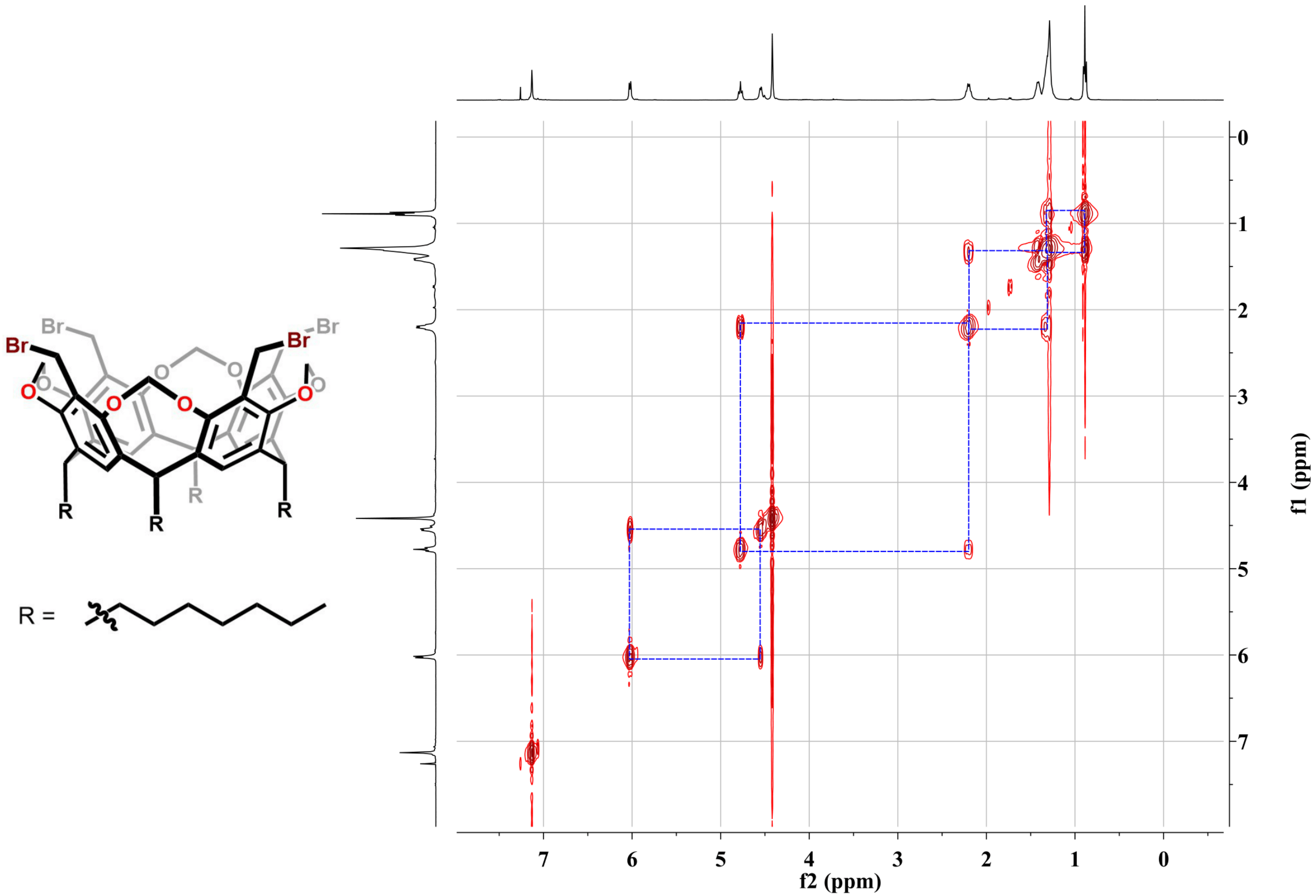

Figure S6. COSY NMR spectrum of **1**. Data collected in $CDCl_3$ at 20 ºC.

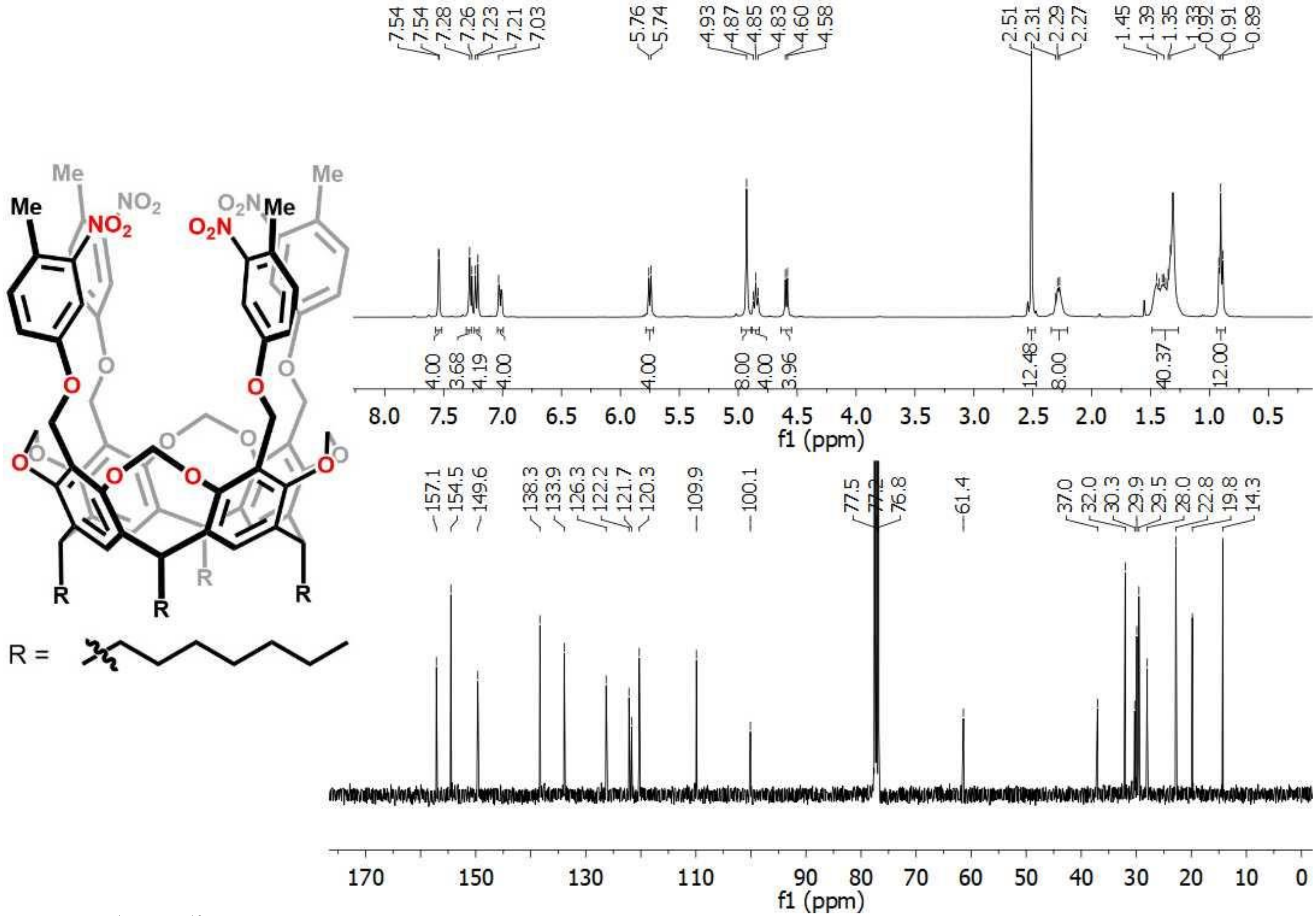

Figure S7. ^{1}H and ^{13}C NMR spectra of **L(NO$_2$)$_4$**. Data collected in $CDCl_3$ at 20 ºC.

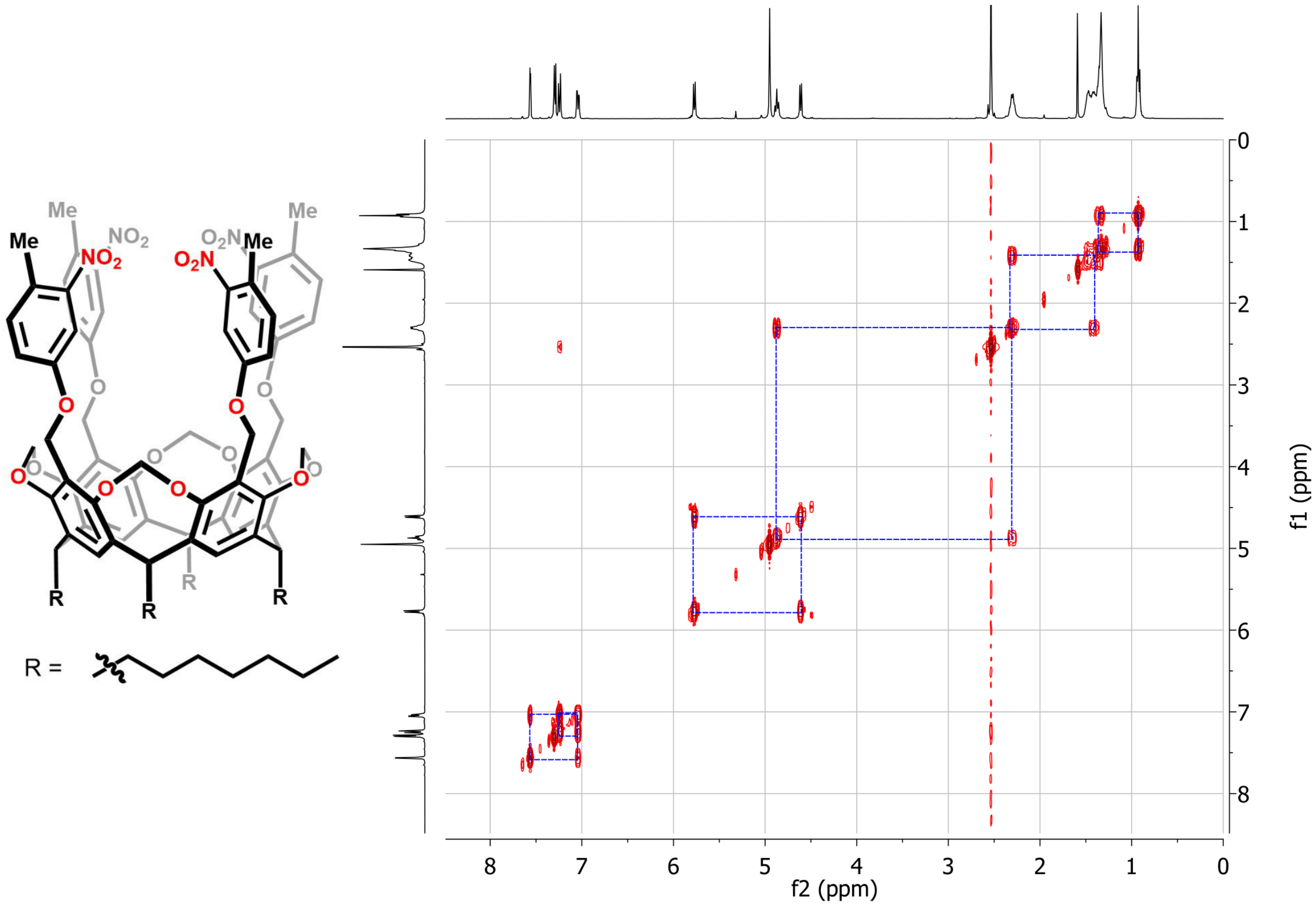

Figure S8. COSY NMR spectrum of **L(NO$_2$)$_4$**. Data collected in $CDCl_3$ at 20 ºC.

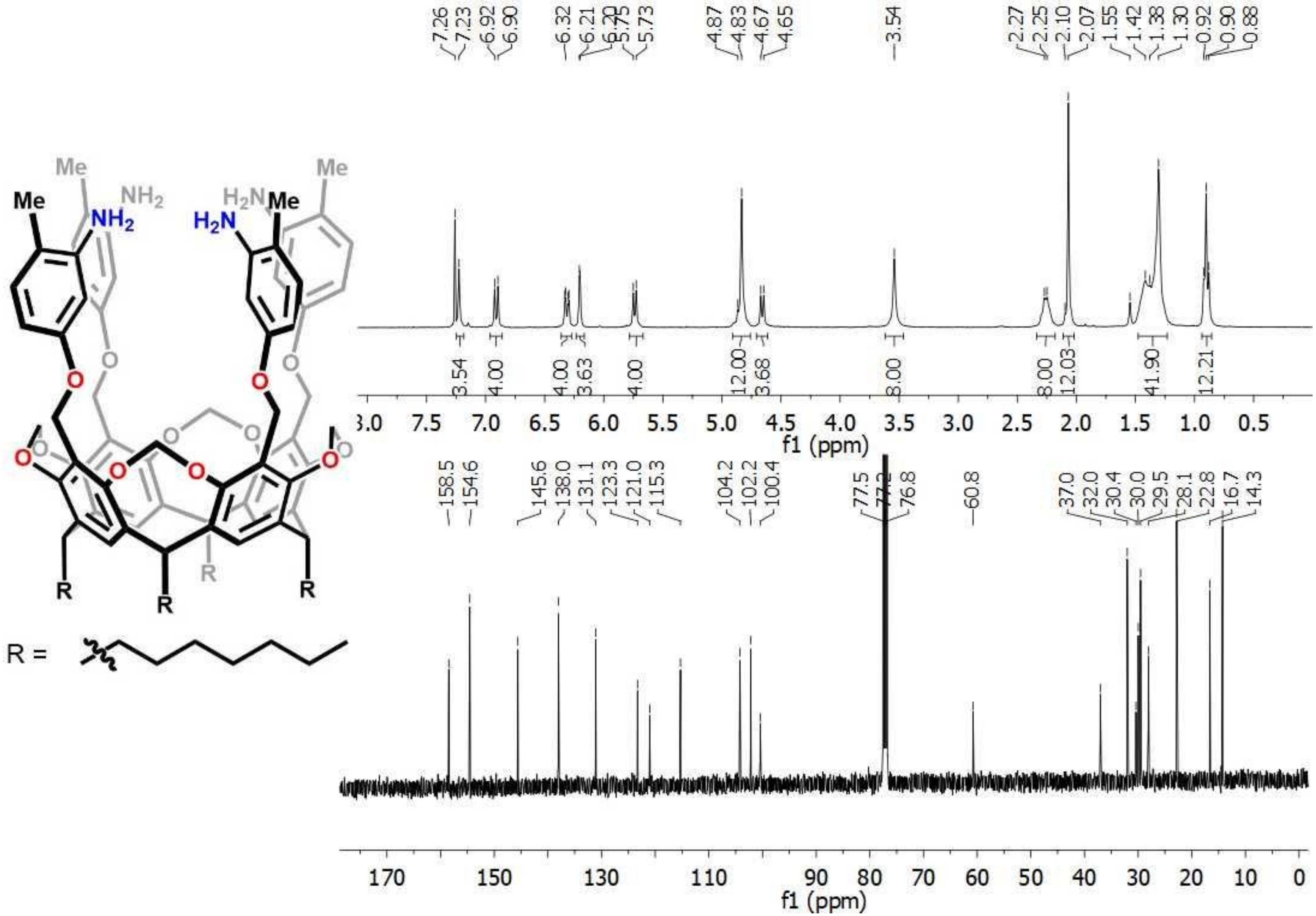

Figure S9. ^{1}H and ^{13}C NMR spectra of **LH_8**. Data collected in $CDCl_3$ at 20 ºC.

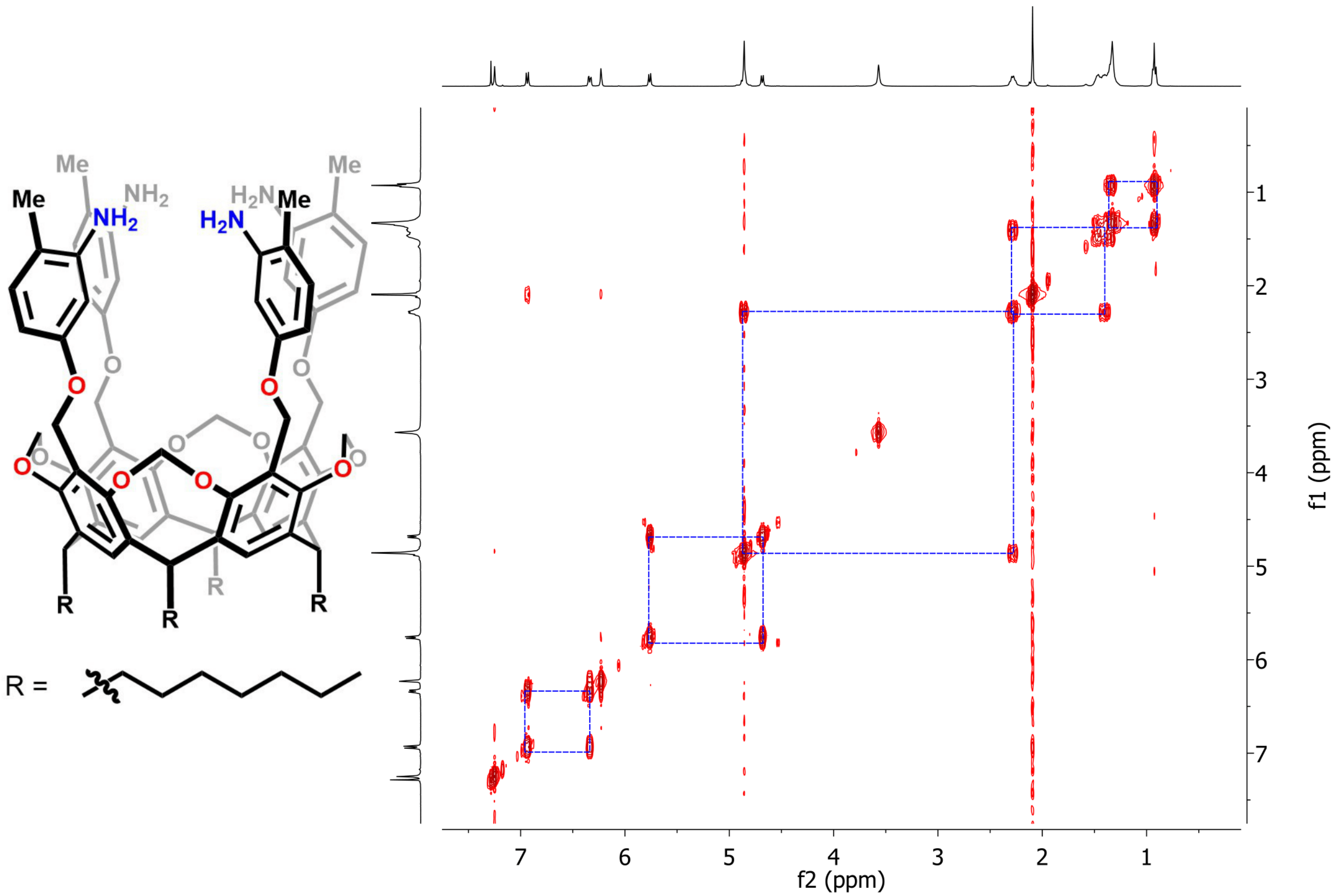

Figure S10. COSY NMR spectrum of **LH₈**. Data collected in $CDCl_3$ at 20 ºC.

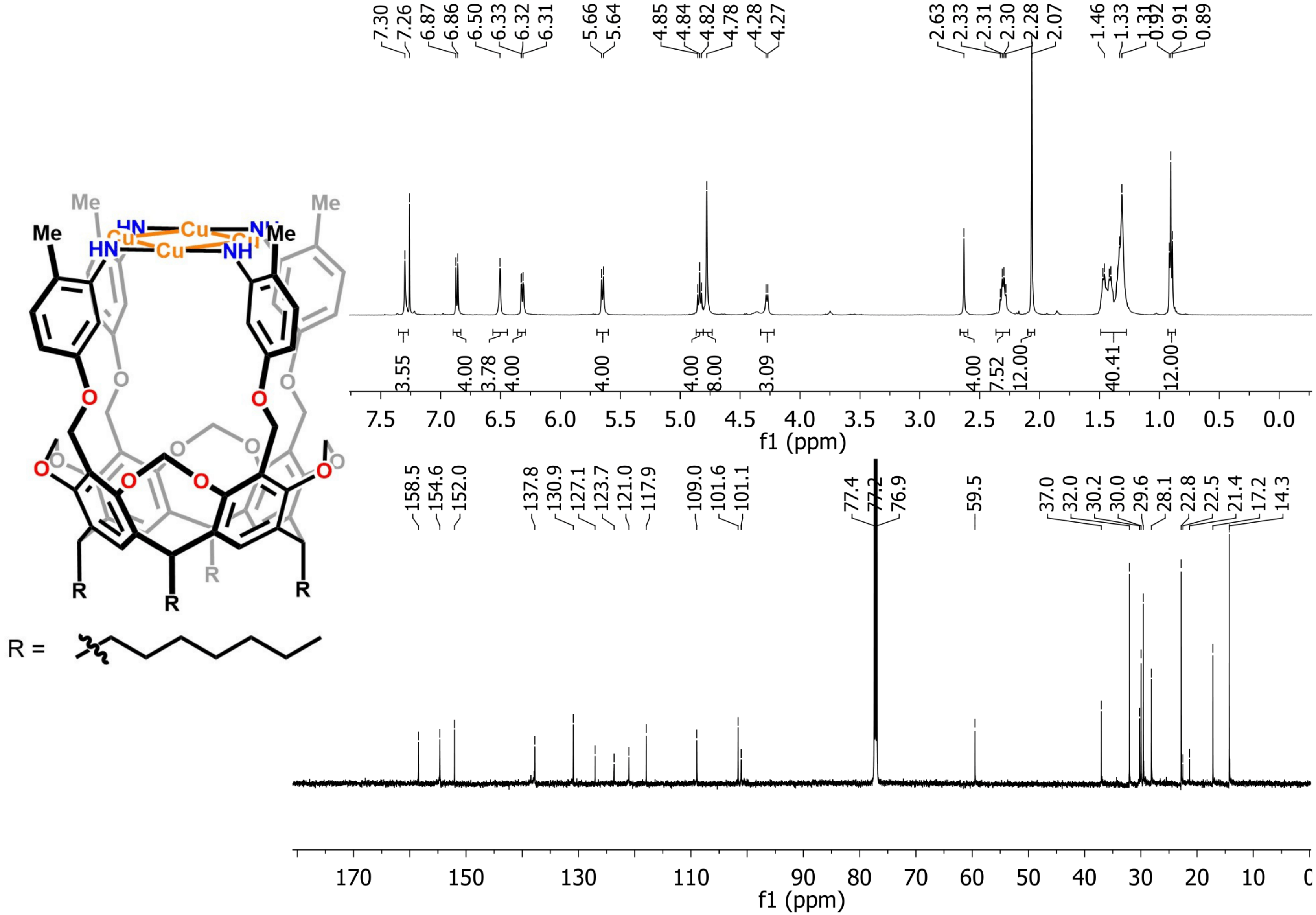

Figure S11. ^{1}H and ^{13}C NMR spectra of $\mathbf{LH_4Cu_4}$. Data collected in $CDCl_3$ at 20 ºC.

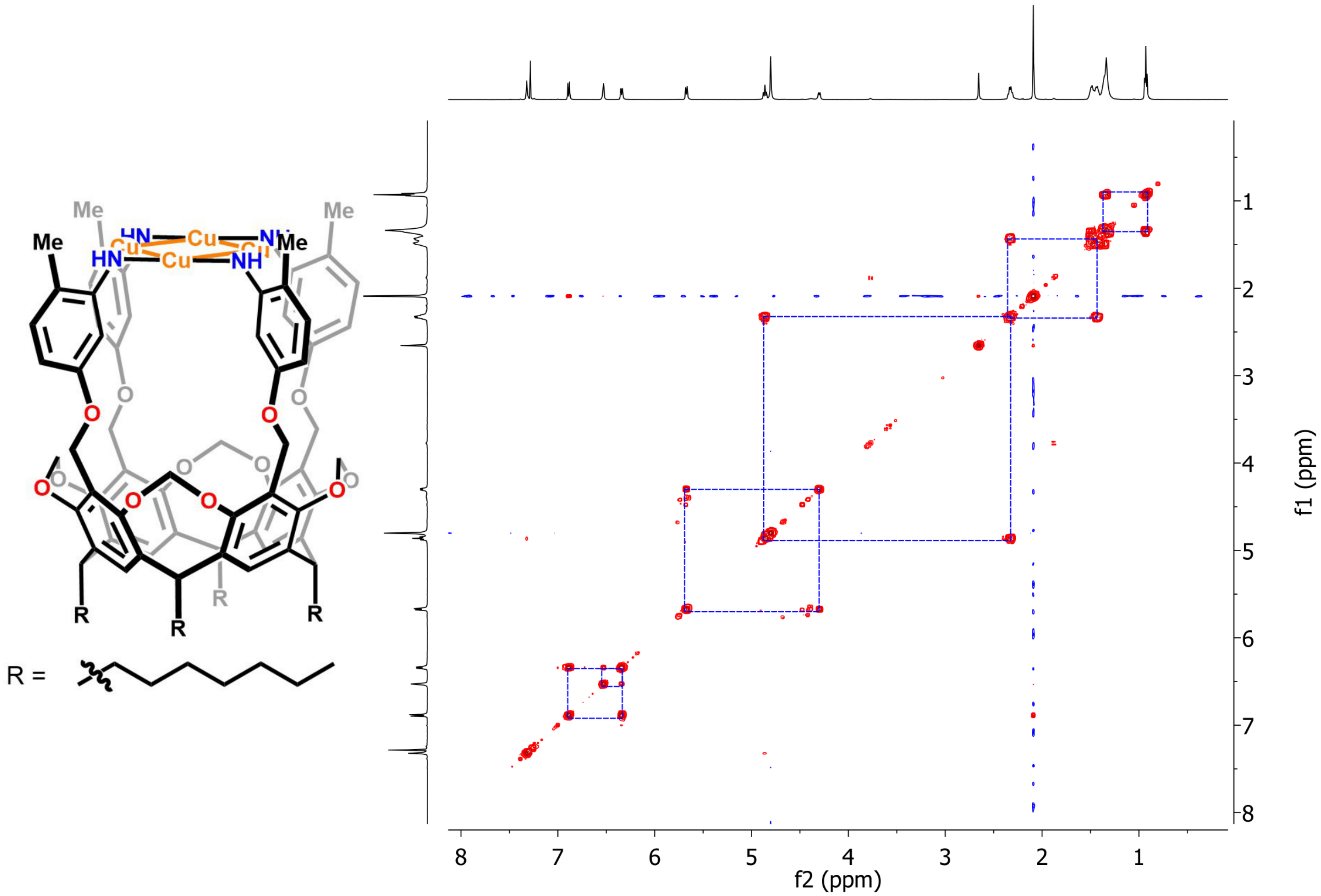

Figure S12. COSY NMR spectrum of $\mathbf{LH_4Cu_4}$. Data collected in $CDCl_3$ at 20 ºC.

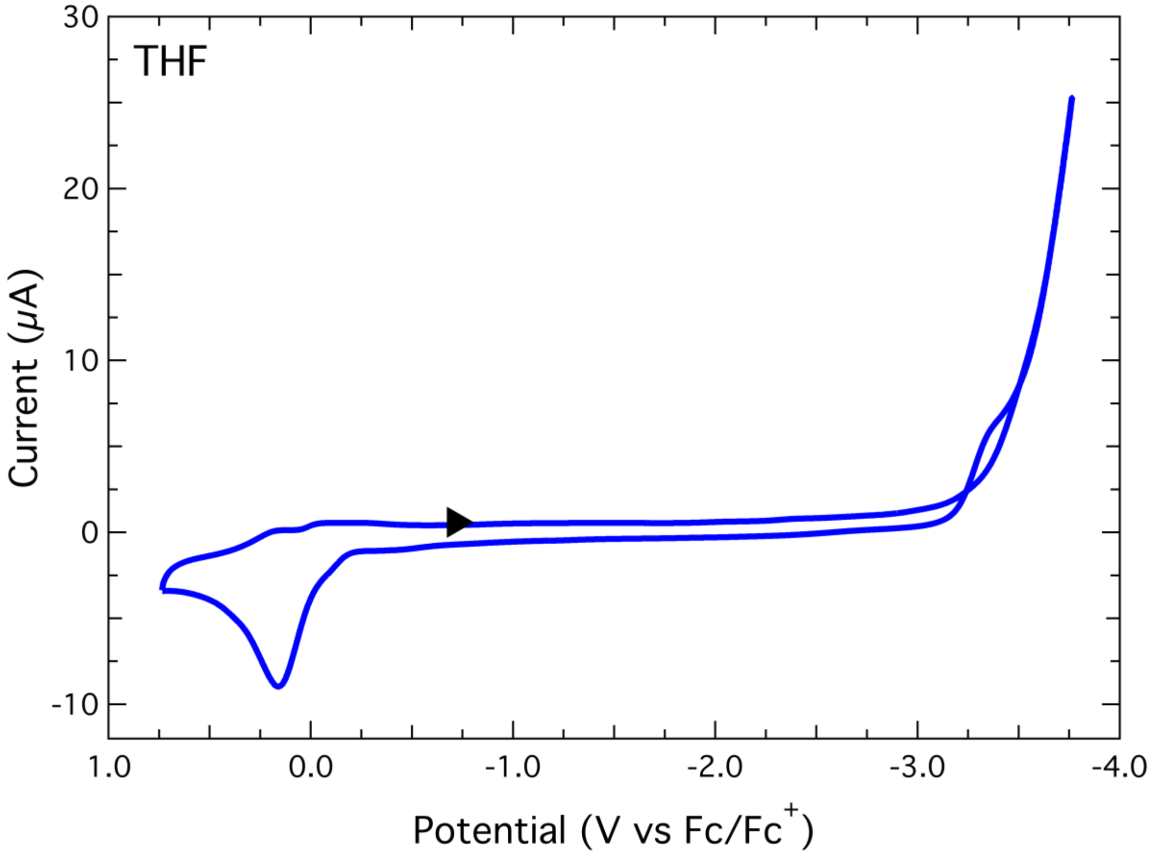

Figure S13. Cyclic voltammetry of **LH_4Cu_4** in THF. A 0.1 M [*n*-Bu_4N][PF_6] solution was used as supporting electrolyte. Scan rate = 50 mV/s.

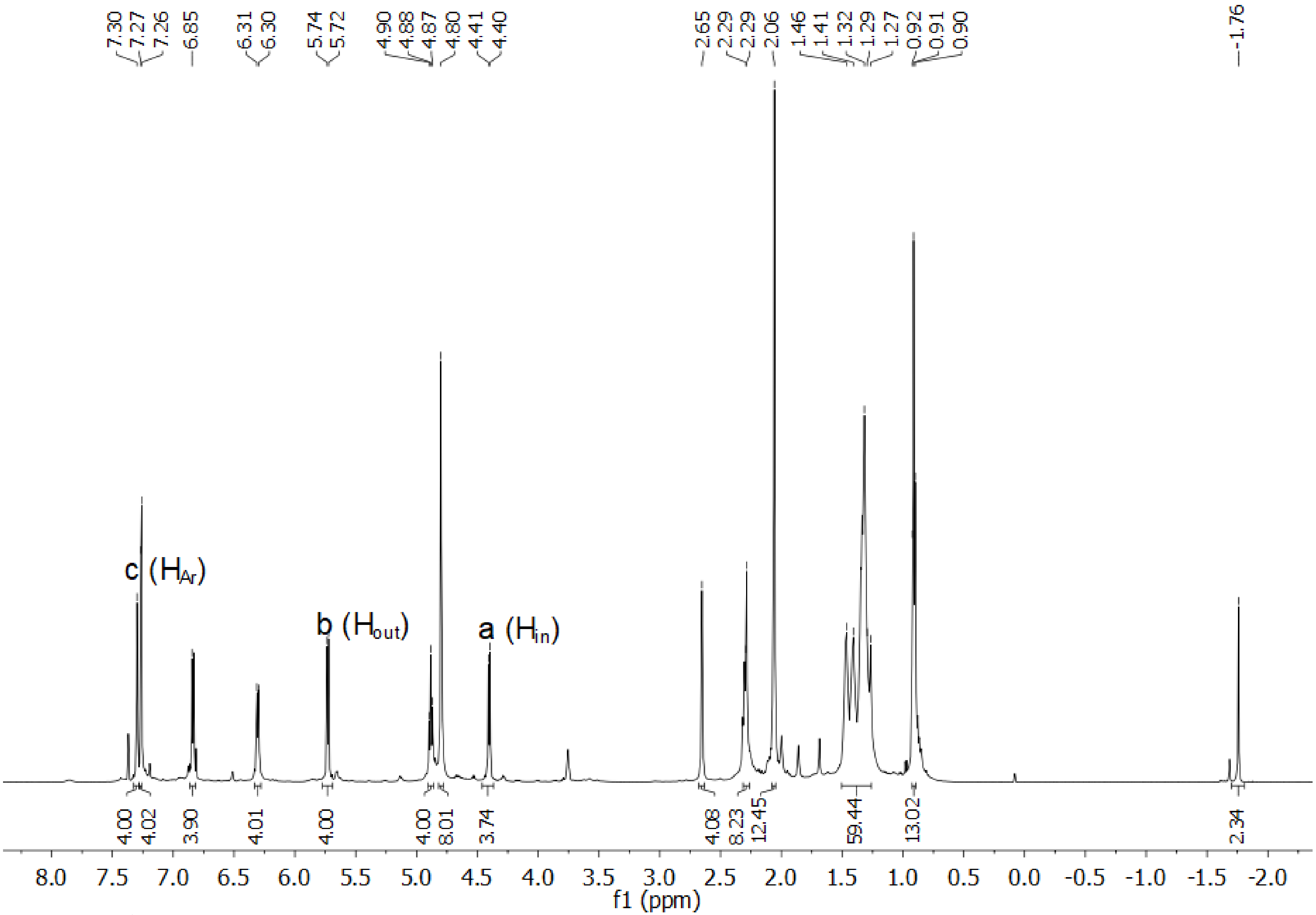

Figure S14. ^{1}H NMR spectrum of **LH_4Cu_4(MeCN)**. Data collected in $CDCl_3$ at 20 ºC.

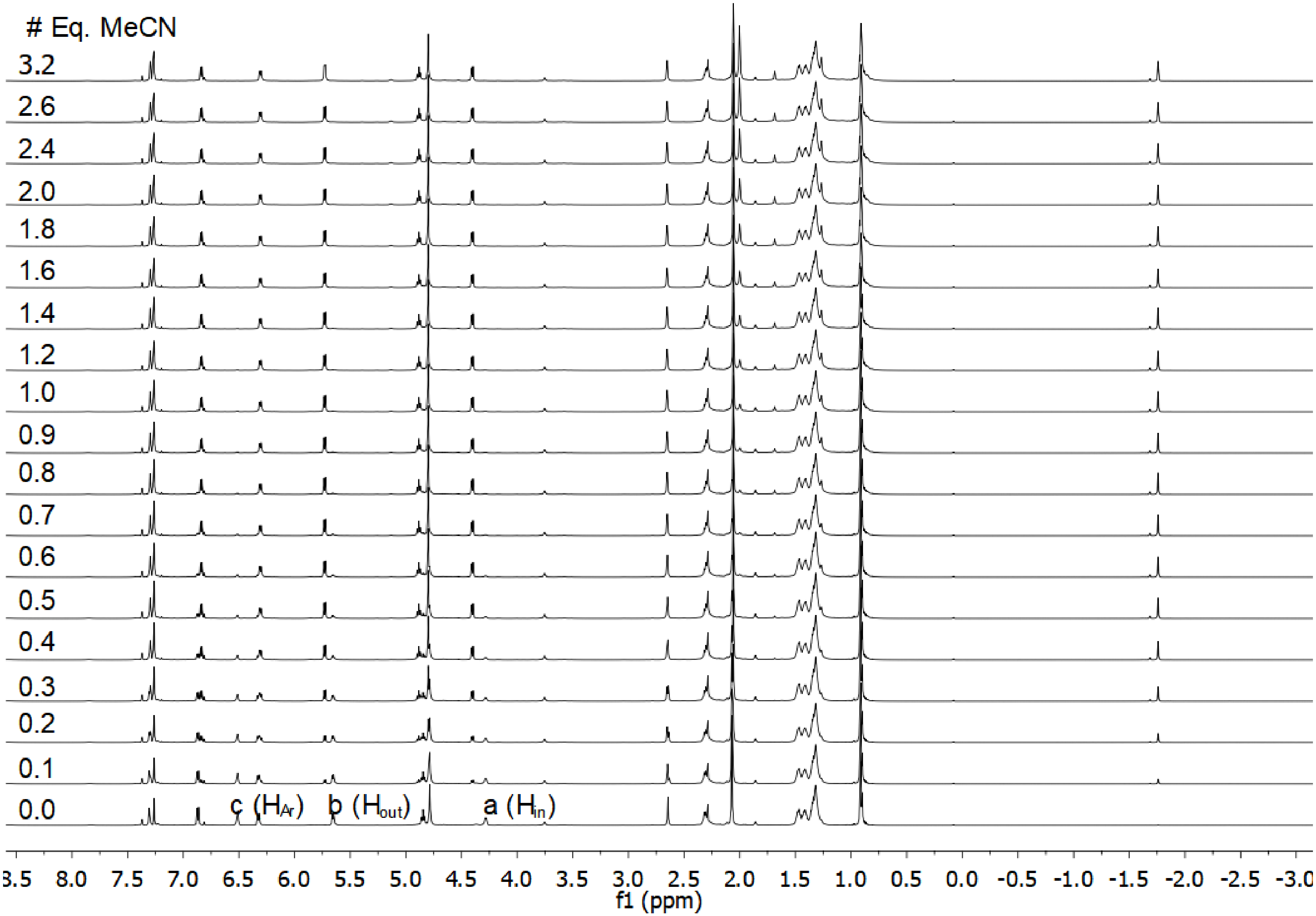

Figure S15. ^{1}H NMR spectra of MeCN titration into **LH_4Cu_4** collected in $CDCl_3$ at 20 ºC.

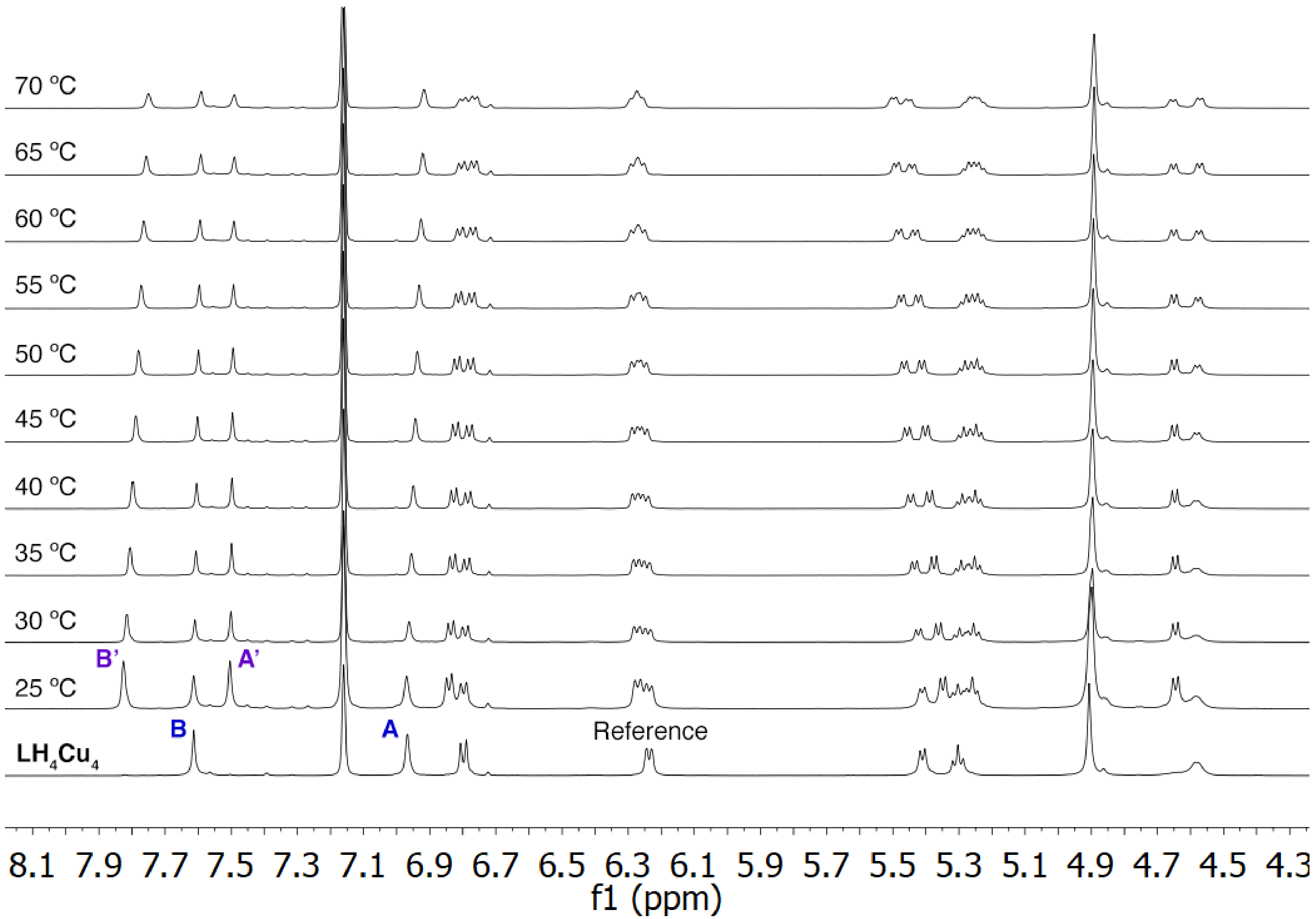

Figure S16. Variable-temperature ^{1}H NMR spectra of MeCN dissociation from **LH_4Cu_4(MeCN)** collected in C_6D_6. As temperature increases, the amount of **LH_4Cu_4** (integration of resonances A and B) increases relative to **LH_4Cu_4(MeCN)** (integration of resonances A' and B').

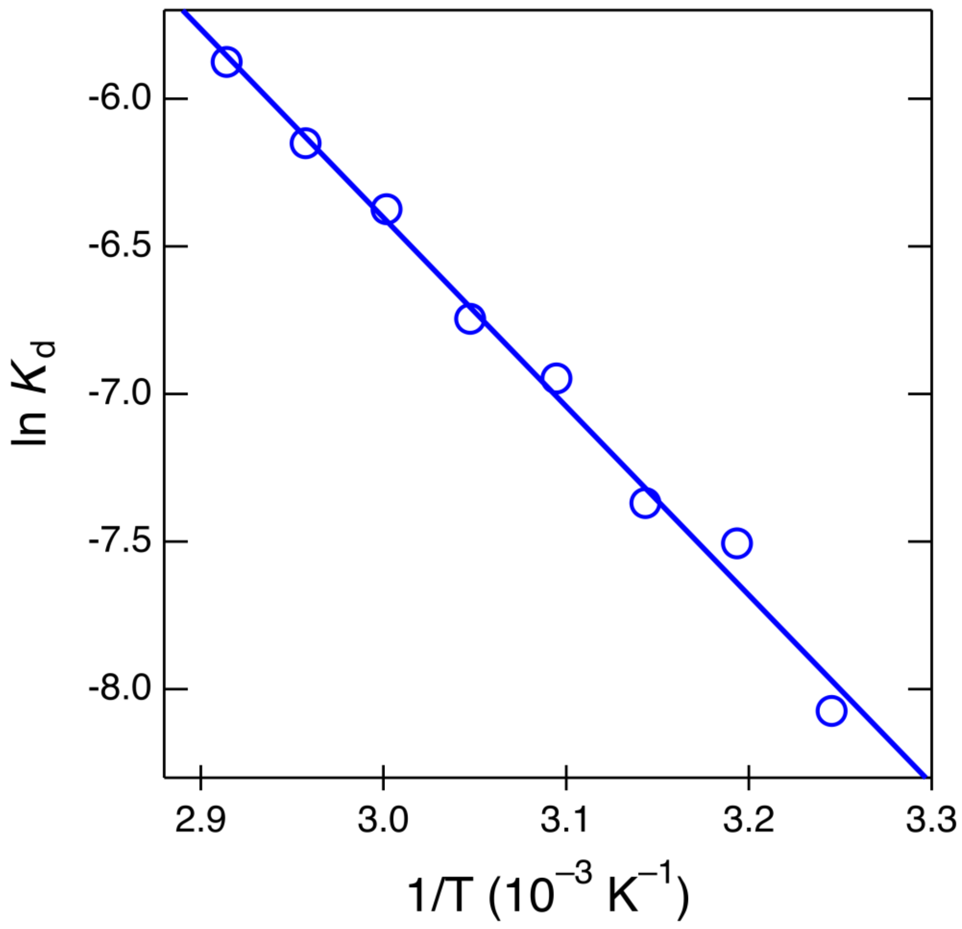

T (ºC)	1/T (K^{-1})	ln K (A/A’)	ln K (B/B’)	ln K (average)
35	0.003245	-7.330	-8.816	-8.073
40	0.003193	-7.148	-7.862	-7.505
45	0.003143	-6.854	-7.882	-7.368
50	0.003094	-6.518	-7.375	-6.947
55	0.003047	-6.613	-6.876	-6.744
60	0.003001	-6.142	-6.604	-6.373
65	0.002957	-5.917	-6.382	-6.150
70	0.002914	-5.737	-6.0122	-5.874

Figure S17. Van’t Hoff analysis[20] of MeCN dissociation from **LH_4Cu_4(MeCN)**. Data from Figure S16 was processed to yield the plot shown here. Average was taken from behavior of resonance A/A’ and B/B’. MeCN dissociation from **LH_4Cu_4(MeCN)** has an enthalpy (ΔH) of 12.7(5) kcal/mol and entropy (ΔS) of 25.4(16) cal/molK.

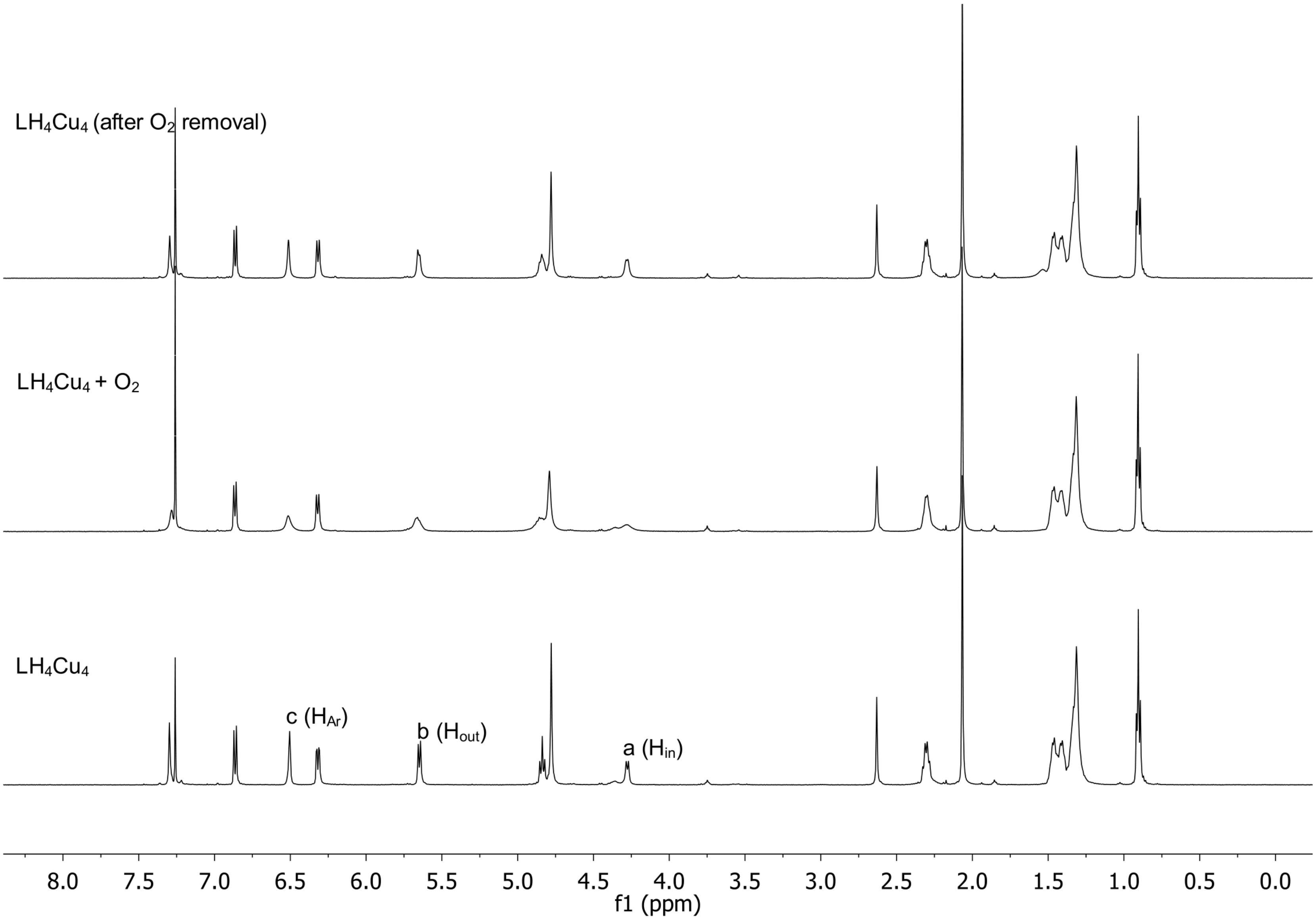

Figure S18. Sequence of ^{1}H NMR spectra as **LH_4Cu_4** is exposed (bottom) first to N_2, (middle) second O_2, and (top) third back to N_2 atmosphere. Data collected in $CDCl_3$ at 20 ºC.

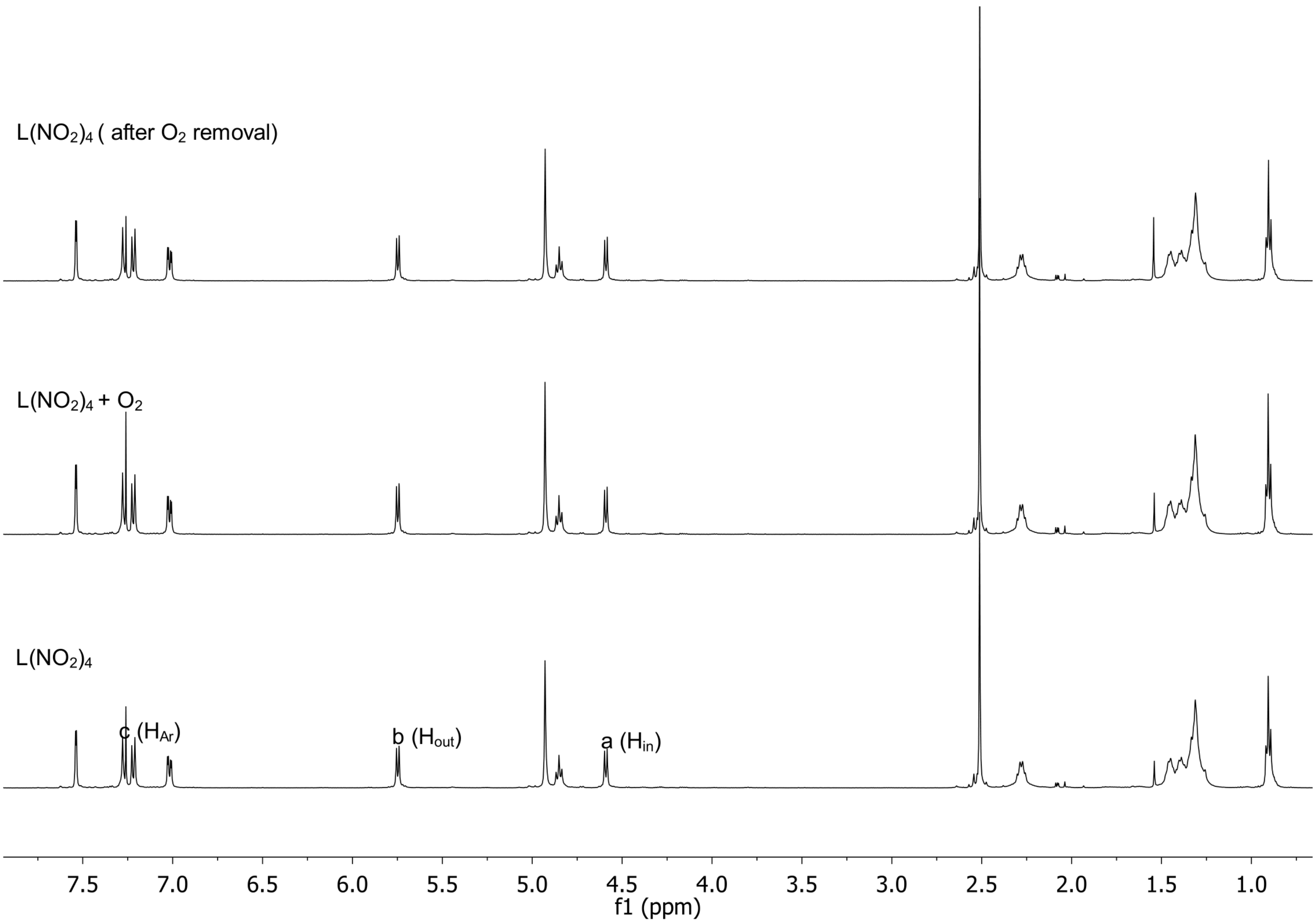

Figure S19. Sequence of ^{1}H NMR spectra as **L(NO$_2$)$_4$** is exposed (bottom) first to N_2, (middle) second O_2, and (top) third back to N_2 atmosphere. Data collected in $CDCl_3$ at 20 ºC.

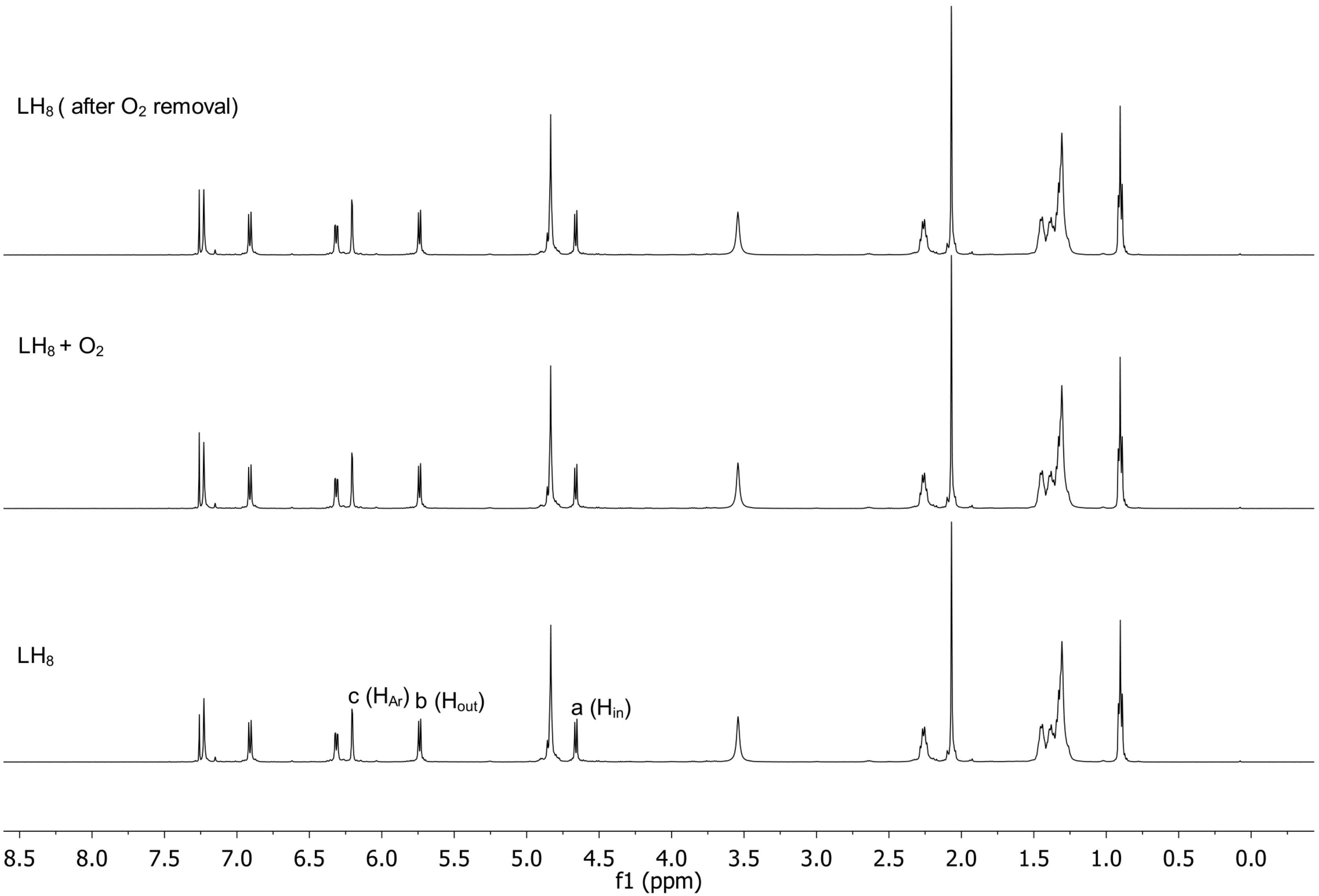

Figure S20. Sequence of ^{1}H NMR spectra as $\mathbf{LH_8}$ is exposed (bottom) first to N_2, (middle) second O_2, and (top) third back to N_2 atmosphere. Data collected in $CDCl_3$ at 20 ºC.

Table S2. Diffusion coefficients extracted from DOSY NMR for **LH_4Cu_4**. Data collected in $CDCl_3$ at 20 ºC.

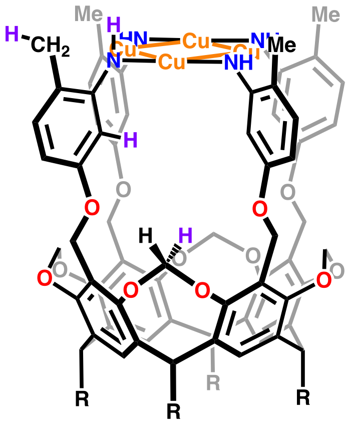

H atom in purple corresponds to the resonance analyzed	Diffusion coefficients (D) from DOSY NMR (× 10^{-6} cm^2/s) in $CDCl_3$
N-H	8.11
C_{Me}-H	7.90
C_{Ar}-H	8.18
C_{CH_2}-H	8.10
Average	8.1(1)

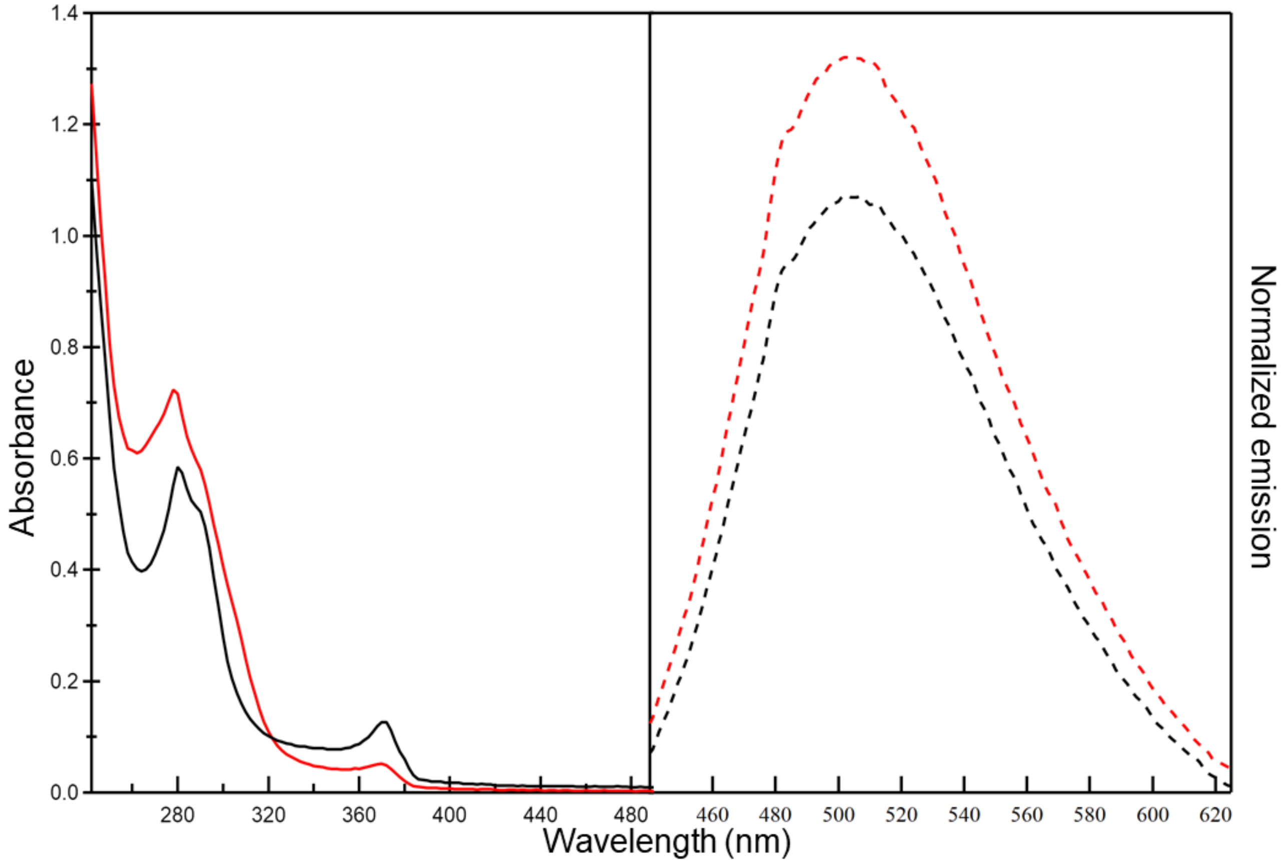

Figure S21. UV-vis and emission spectra of **LH_4Cu_4** under N_2 (red trace) and **LH_4Cu_4** under O_2 (black trace) at room temperature in $CHCl_3$. Excitation at 420 nm was used to generate the emission spectra.

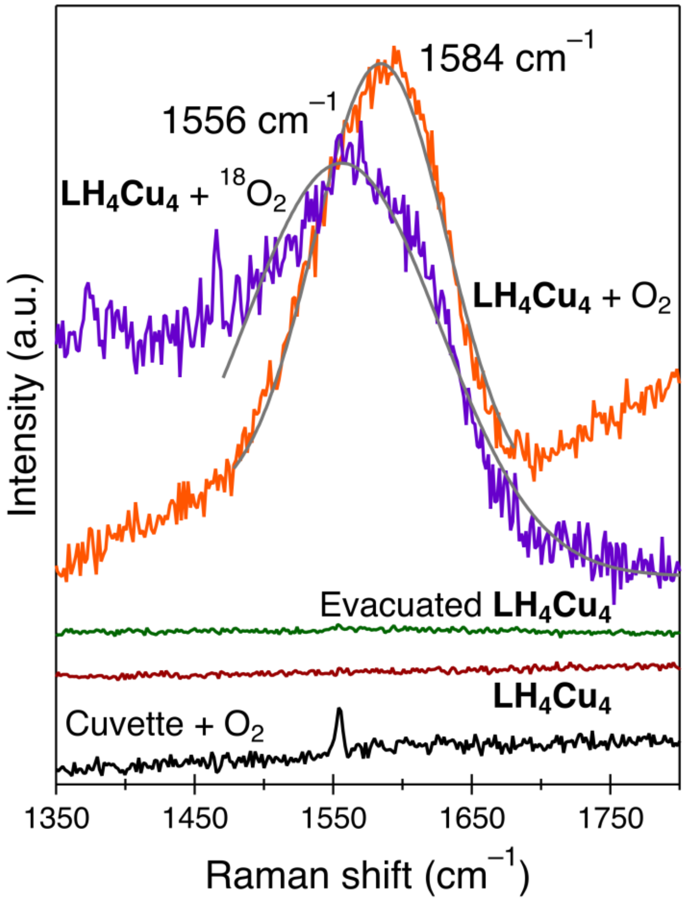

Figure S22. Variable isotope resonance Raman spectra of **LH_4Cu_4** plus O_2 collected at room temperature. The region expected for the O–O stretch is shown. Partial fits (in grey) of the orange and purple traces to gaussian curves served to locate the maximum. Data collection sequence: 1) First, the spectrum of **LH_4Cu_4** was collected at the beginning of the experiment and prior to O_2 introduction (brown trace), 2) Dioxygen was introduced in the cuvette and the spectrum was recorded (orange trace), and 3) the cuvette's atmosphere was removed under high vacuum (green trace). This collection sequence was executed at least eight times with different batches of **LH_4Cu_4**. Experiments with $^{18}O_2$ followed steps 1-3 (with $^{16}O_2$) described before, and subsequently in step 4) $^{18}O_2$ was introduced and the spectrum was recorded, and last 5) the cuvette was evacuated and the spectrum recorded again. As a control, data was collected for hours in the absence of **LH_4Cu_4** simply filling the cuvette with O_2 (black trace).

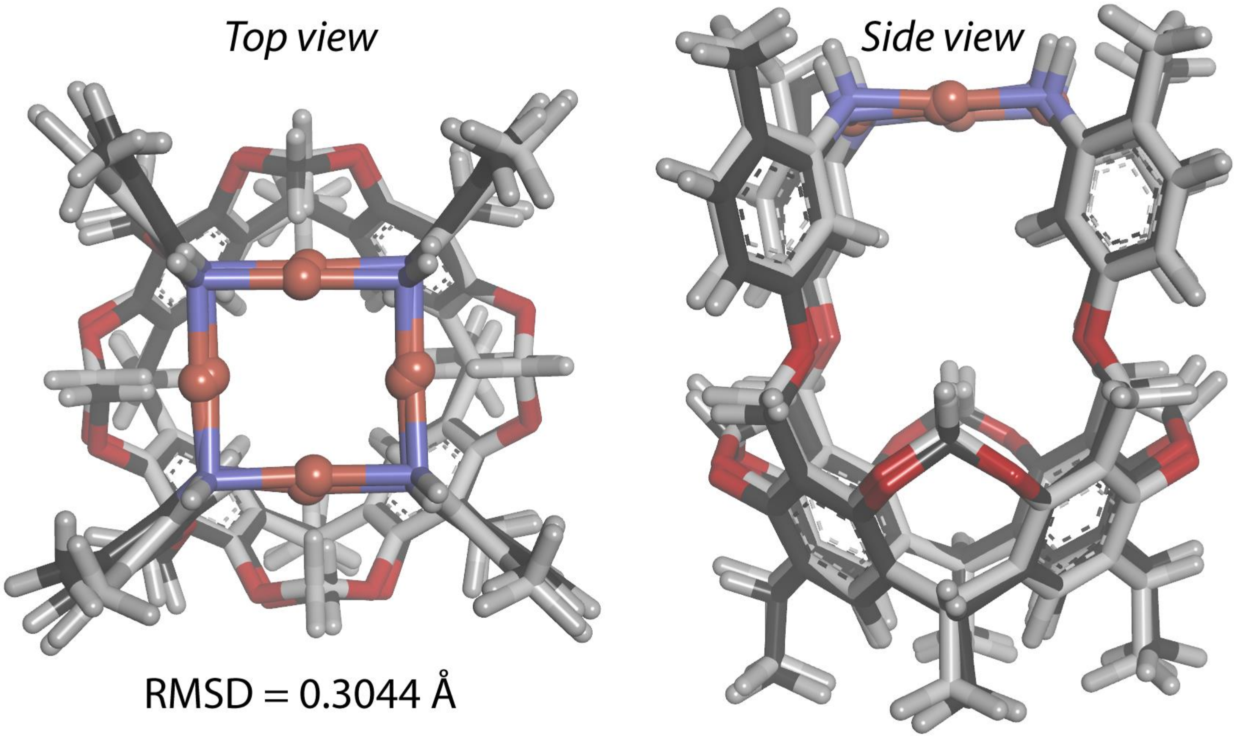

Figure S23. Heavy atom root mean square deviation (RMSD) between the molecular crystal structure of $\mathbf{LH_4Cu_4}$ (black bonds and carbons) and DFT-calculated structure $\mathbf{LH_4Cu_4'}$ (light grey bonds and carbons). For this analysis R^3 in the crystal structure was truncated to a methyl.

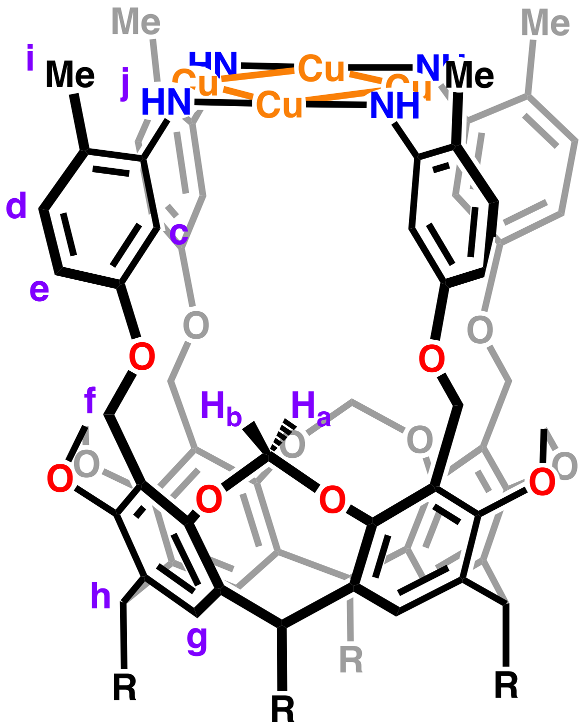

Table S3. DFT Calculated ^{1}H NMR data of **LH_4Cu_4'** (R = Me) using B3LYP-D3BJ/BS+PCM($CHCl_3$) level of theory.

	Def2-SVP	Def2-TZVP	**Exp.**
a	4.62	4.51	4.28
b	5.75	5.67	5.65
c	7.20	7.17	6.50
d	7.12	7.08	6.86
e	6.35	6.39	6.32
f	4.95	4.82	4.78
g	8.14	7.84	7.30
h	5.26	5.14	4.84
i	2.39	2.54	2.07
j	2.77	2.69	2.63

Cartesian Coordinates of optimized molecules (B3LYP-D3BJ/BS+PCM($CHCl_3$))

$^{Me}LCu_4$'
# B3LYP-D3BJ/Def2-SVP+PCM($CHCl_3$)

Zero-point correction= 1.211955 (Hartree/Particle)
Thermal correction to Energy= 1.288199
Thermal correction to Enthalpy= 1.289143
Thermal correction to Gibbs Free Energy= 1.103781
Sum of electronic and zero-point Energies= -10309.570135
Sum of electronic and thermal Energies= -10309.493891
Sum of electronic and thermal Enthalpies= -10309.492947
Sum of electronic and thermal Free Energies= -10309.678309

0 1

O	1.06175800	4.55539000	4.21438400
O	-1.28393800	4.52685900	4.14439900
O	-4.61052400	1.11971300	4.10693300
O	-4.58280300	-1.22671100	4.15048700
O	-1.17922500	-4.55320500	4.31355800
O	1.16654100	-4.52510900	4.38489700
O	4.49248500	-1.11725900	4.41954600
O	4.46566500	1.22918000	4.37523300
C	2.75035800	2.87834800	4.29105200
C	1.61637000	3.47305000	4.86194600
C	-0.08614000	4.34499200	3.42236800
C	-1.84924500	3.43073700	4.75858300
C	-2.93287400	2.80931400	4.12210900
C	-3.53518700	1.70415000	4.73968800
C	-4.39169700	-0.06507400	3.37376300
C	-3.49461100	-1.76244400	4.80413000
C	-2.86669800	-2.87573800	4.22806500
C	-1.76956200	-3.44823300	4.88694700
C	0.01484300	-4.37118800	3.58543100
C	1.69582900	-3.40678200	4.99117700
C	2.81582100	-2.80767700	4.39781700
C	3.38145600	-1.67984900	5.00900700
C	4.31835000	0.03967800	3.63190100
C	3.34156200	1.78742300	4.94360100
C	1.71823700	1.93897300	6.70441700
C	1.08787600	3.02136900	6.08167200
C	-0.17349000	3.66626000	6.64265400
C	-1.38307900	2.99119800	6.00786000
C	-2.02302100	1.89373000	6.59326300
C	-3.09845300	1.23456700	5.98860500
C	-3.75104000	0.00199800	6.60219100
C	-3.06943300	-1.23695600	6.03466100

C	-1.97924000	-1.84806500	6.66287300
C	-1.31410300	-2.95179000	6.11871400
C	-0.08987100	-3.57478700	6.77822600
C	1.15675200	-2.92229600	6.19362900
C	1.76203600	-1.80355300	6.77569200
C	2.87192500	-1.16546500	6.21190100
C	3.48830000	0.08972500	6.81699600
C	2.84312200	1.30636700	6.16480800
C	-0.21958800	3.70198000	8.17177000
C	-3.80512200	0.02995100	8.13121800
C	-0.13670900	-3.55443500	8.30755300
H	-0.07271000	5.12854200	2.65427600
H	-3.38601900	-0.06143600	2.92341700
H	-5.16617100	-0.08867500	2.59676300
H	0.01815200	-3.38867000	3.08656400
H	0.04700200	-5.18266300	2.84748100
H	5.13671400	0.03518800	2.90089900
H	3.34047600	0.01913600	3.12435200
H	1.31133400	1.56852000	7.64493300
H	-0.17528200	4.70383600	6.27954500
H	-1.66402000	1.53246600	7.55656400
H	-4.78422000	-0.01715100	6.22726600
H	-1.62893400	-1.44280500	7.61174800
H	-0.06744000	-4.62490600	6.45390200
H	1.34717300	-1.40813400	7.70254000
H	4.54192600	0.09599200	6.50350500
H	-0.22121100	2.70065100	8.62678600
H	-2.80937900	0.05105800	8.59786700
H	-4.32633100	-0.86215400	8.50917600
H	-0.16267400	-2.53701600	8.72449900
H	0.75017300	-4.05686900	8.72150800
C	3.45089000	0.11845500	8.34643300
H	3.97059300	-0.76058900	8.75576200
H	3.95020000	1.02409200	8.72174900
H	2.42874100	0.11453500	8.75242700
H	0.65521400	4.24048000	8.56560900
H	-4.34835000	0.92247100	8.47572300
H	-1.03394300	-4.07985300	8.66709700
H	-0.06038300	3.34467000	2.96091000
H	-1.12894000	4.21912400	8.51236700
C	3.02992100	3.00236000	0.65009800
C	3.98557500	3.97941700	0.33332900
C	2.39036800	2.29382700	-0.37346000
C	4.27400900	4.22073600	-1.01368900
H	4.50179700	4.54786000	1.10428200
C	2.68705300	2.54307400	-1.72183600

C	3.65181900	3.53021500	-2.05505900
H	5.01858100	4.98267200	-1.26171000
O	2.66603800	2.68146500	1.92505800
C	3.30396200	3.38330400	2.99382400
H	3.10743500	4.46187400	2.89854100
H	4.38979500	3.20801000	2.95633200
C	-3.34421100	-3.42783100	2.91980900
H	-3.14640800	-4.50955200	2.87504400
H	-4.42529100	-3.25232600	2.81307500
C	-3.42330600	3.30222300	2.79499600
H	-4.50102300	3.10211400	2.69709200
H	-3.24651600	4.38507900	2.70961300
C	3.38169000	-3.34665000	3.11971200
H	4.46339400	-3.14874200	3.07662600
H	3.20926600	-4.43204700	3.06256300
O	2.72963900	-2.69733800	2.02659800
O	-2.64266400	-2.76653400	1.86512700
O	-2.71090200	2.61560500	1.76405300
C	3.10513900	-3.05112300	0.76378000
C	4.08883600	-4.01077500	0.48166400
C	2.44772500	-2.39634700	-0.28399300
C	4.38736600	-4.28888300	-0.85604500
H	4.61897700	-4.53803000	1.27219800
C	2.75442300	-2.68245700	-1.62276400
C	3.74810800	-3.65199400	-1.92099800
H	5.15406100	-5.03704600	-1.07713800
C	-2.94447000	-3.12214900	0.58319200
C	-3.90124800	-4.09141500	0.24643600
C	-2.23761300	-2.45901200	-0.42644100
C	-4.12522600	-4.36848900	-1.10594400
H	-4.46735400	-4.62658300	1.00609600
C	-2.46979200	-2.74405800	-1.78038500
C	-3.43682600	-3.72189000	-2.13375600
H	-4.87156000	-5.12347200	-1.36944100
C	-3.01487400	2.92390200	0.47039700
C	-3.98176500	3.87042900	0.09967400
C	-2.29910500	2.23482300	-0.51530300
C	-4.20486200	4.10049100	-1.26164100
H	-4.55595000	4.42384800	0.83994000
C	-2.53033700	2.47269800	-1.87849700
C	-3.50639400	3.42837800	-2.26605900
H	-4.95845500	4.83835700	-1.55161000
H	-1.55045900	1.50242600	-0.20580700
H	-1.49528600	-1.70956900	-0.14328700
H	1.68538900	-1.65240500	-0.04262100
H	1.65024400	1.53669300	-0.10543800

N	2.02944200	1.81603100	-2.73347800
H	2.35824000	2.10708200	-3.65155200
N	2.07814800	-2.00988000	-2.65943500
H	2.41358400	-2.32633200	-3.56664500
N	-1.74635400	-2.06100200	-2.77768000
H	-2.02028400	-2.38684900	-3.70204500
N	-1.79578800	1.76586200	-2.85092500
H	-2.07770100	2.05057800	-3.78642400
Cu	2.02450900	-0.09581700	-2.62397300
Cu	0.11496500	1.76675300	-2.72206400
Cu	-1.74656600	-0.14590700	-2.74504100
Cu	0.16316800	-2.00817800	-2.64691100
C	3.98521100	3.81479400	-3.49604000
H	4.37911600	2.92089300	-4.01270800
H	3.09949200	4.14946700	-4.06580600
H	4.74630700	4.60363700	-3.57705700
C	4.09310800	-3.97515000	-3.35106900
H	3.21914200	-4.35548800	-3.91023400
H	4.46114000	-3.08779900	-3.89726100
H	4.87762100	-4.74313400	-3.40440000
C	-3.70375600	-4.04223700	-3.58105700
H	-4.04936900	-3.15533800	-4.14240300
H	-2.79899200	-4.41392400	-4.09530400
H	-4.47841900	-4.81593500	-3.67800700
C	-3.77096100	3.70045800	-3.72362700
H	-2.86743300	4.06373400	-4.24597200
H	-4.10677400	2.79347900	-4.25818200
H	-4.55206400	4.46377400	-3.84751600

$^{Me}LCu_4'(O_2)$
B3LYP-D3BJ/Def2-TZVP+PCM($CHCl_3$)

Zero-point correction= 1.216794 (Hartree/Particle)
Thermal correction to Energy= 1.297105
Thermal correction to Enthalpy= 1.298049
Thermal correction to Gibbs Free Energy= 1.100792
Sum of electronic and zero-point Energies= -10459.784170
Sum of electronic and thermal Energies= -10459.703859
Sum of electronic and thermal Enthalpies= -10459.702915
Sum of electronic and thermal Free Energies= -10459.900172

0 3

Cu	10.71376900	25.31132800	17.65954300
Cu	8.94335400	24.87304900	15.71688700
Cu	10.77739300	23.51356100	14.34377500
Cu	12.54995600	23.94954200	16.28783300
O	14.57462800	28.91835800	16.72303100
O	9.40947800	30.22328100	15.90534800
O	9.52130600	27.66955600	11.17860400
O	14.66273300	26.36378700	11.99441000
O	13.75169800	32.17336800	17.06699300
O	8.34103700	31.96174800	13.24202400
O	11.67001000	28.43594500	8.72395500
O	17.07630300	28.64159500	12.55180500
O	11.50386900	32.73964400	16.71176000
O	8.38396200	30.84967300	11.17831200
O	13.91596600	27.86381100	9.07993900
O	17.03758500	29.75860900	14.61317600
N	12.52486900	24.79485800	18.00567900
H	12.54619500	24.08600100	18.73564300
N	8.85879300	25.72591200	17.42920700
H	8.30045800	25.16134200	18.06583000
N	8.92503800	23.90611000	14.06354900
H	8.37335300	23.05716000	14.16654300
N	12.58907300	22.97008300	14.64247400
H	12.62441800	21.97130900	14.83449100
C	13.46980400	25.80489400	18.27225000
C	8.46045300	27.07560900	17.48898300
C	8.55124100	24.59923700	12.89581600
C	13.55650500	23.32376300	13.68162600
C	14.29293000	25.73439100	19.42707400
C	7.41505900	27.48019100	18.36059200
C	7.52618400	24.09350600	12.05365200
C	14.41289600	22.33921400	13.12228100
C	15.20978800	26.76382300	19.64609100

H	15.84719100	26.71744500	20.53378000
C	7.06049300	28.83002900	18.38137300
H	6.25605900	29.15003000	19.04989700
C	7.19374500	24.82293600	10.91093100
H	6.40510000	24.44057700	10.25649900
C	15.35321400	22.74677900	12.17444900
H	16.01691700	21.99459500	11.73836300
C	15.34741700	27.85197600	18.77847800
H	16.07860000	28.62690900	18.99919700
C	7.69081300	29.79044600	17.58384300
H	7.37328000	30.82957500	17.64101300
C	7.82590200	26.02239600	10.56805100
H	7.52543700	26.54966000	9.66497700
C	15.48251200	24.07591400	11.75896800
H	16.23361000	24.33672200	11.01619500
C	14.52994100	27.90903500	17.63985200
C	8.72258900	29.37831400	16.72725700
C	8.83686800	26.51192300	11.40867000
C	14.63046000	25.03931200	12.31944900
C	13.60166100	26.89117800	17.39429400
H	12.97587500	26.95665700	16.50231700
C	9.09897700	28.03119300	16.68439500
H	9.90374200	27.73221700	16.01019300
C	9.19242400	25.80106100	12.56022800
H	9.98155700	26.19818100	13.20112800
C	13.67795900	24.65970500	13.27135300
H	13.02503700	25.42457900	13.69490600
C	14.16998800	24.57581200	20.38151800
H	13.15574600	24.50358800	20.81421200
H	14.87775400	24.67505100	21.21658300
H	14.37589200	23.61000400	19.88563200
C	6.71675500	26.47126200	19.23398100
H	6.23042900	25.67710500	18.63909200
H	5.93758400	26.94928500	19.84431100
H	7.41768400	25.96950900	19.92538100
C	6.82387500	22.80588900	12.39597900
H	7.52566800	21.95375300	12.44514300
H	6.06019900	22.56053100	11.64449400
H	6.31772800	22.86071400	13.37679000
C	14.29993600	20.89911500	13.54839900
H	14.47642000	20.77504800	14.63215700
H	15.03317200	20.27242700	13.02137100
H	13.29758600	20.48433500	13.33783800
C	15.50362000	29.97975300	16.95123800
H	16.52666700	29.57583000	16.98667600
H	15.27521700	30.47301900	17.90807100

C	9.05480500	31.60721900	15.93231900
H	9.19752800	32.00678700	16.94762000
H	8.00012700	31.72524100	15.64115100
C	9.17759700	28.42067000	10.01259400
H	8.11724200	28.71123400	10.05780300
H	9.34753800	27.80820100	9.11417200
C	15.62571200	26.78431100	11.02663700
H	15.44942700	26.25878100	10.07596300
H	16.63914400	26.55160200	11.38697600
C	15.38423400	30.96350500	15.82817900
C	9.93477500	32.34087700	14.96749800
C	10.03910000	29.64489700	9.96483400
C	15.48443600	28.26167200	10.82457100
C	16.15129600	30.81198000	14.66514200
C	11.16156200	32.87535400	15.38420300
C	9.62184500	30.83619800	10.57393900
C	14.60854900	28.76819600	9.85476300
C	16.06151200	31.72602900	13.60357700
C	11.99640200	33.57228300	14.49619700
C	10.41064100	31.99676900	10.53018700
C	14.47424800	30.14877000	9.63829600
C	15.16122300	32.78897500	13.73318400
H	15.06874100	33.49836700	12.91114700
C	11.57401500	33.69983700	13.16863800
H	12.21753600	34.22601000	12.46404400
C	11.64406800	31.92227800	9.87432700
H	12.27409700	32.81094200	9.84492700
C	15.23040700	31.01056900	10.43975300
H	15.12740000	32.08538700	10.29290500
C	14.36597900	32.97179700	14.86952300
C	10.36473500	33.17047800	12.70556900
C	12.10642300	30.75141400	9.26414500
C	16.10554300	30.55022200	11.42922000
C	14.48531800	32.03568400	15.90910700
C	9.55816000	32.48119300	13.62486100
C	11.28793700	29.61274700	9.32975300
C	16.21095600	29.16263100	11.61498700
C	12.41530900	31.72307400	17.06467100
H	12.18306700	31.45294200	18.10260700
H	12.30153300	30.85226400	16.39873300
C	8.32737000	30.71624300	12.58104900
H	9.14921900	30.07706600	12.94234700
H	7.35345600	30.26294300	12.80509300
C	12.57143000	27.59680500	9.41106900
H	12.36756400	26.57743800	9.05967100
H	12.41808700	27.67403900	10.49951300

C	16.65645900	28.60464400	13.89753500
H	17.19287900	27.76717100	14.36127300
H	15.56653200	28.45403900	13.95951100
C	13.34305500	34.09580400	14.98010300
H	13.22530300	34.29864400	16.05409600
C	9.93931800	33.25718800	11.24506500
H	8.84067500	33.21847600	11.24284500
C	13.47687000	30.65776800	8.60504700
H	13.39695100	29.87062100	7.84181700
C	16.87995000	31.49540600	12.33939500
H	17.78113300	30.94953200	12.65294400
C	13.77966200	35.39512000	14.29999700
H	13.92300400	35.28657800	13.21497300
H	13.02234600	36.17798600	14.45478600
C	10.36655500	34.55396500	10.55411500
H	9.95923900	35.42295800	11.09197600
H	11.45833400	34.67868300	10.50732600
C	13.91555300	31.94732300	7.90787600
H	13.17754900	32.23673000	7.14501600
H	14.02390100	32.79611200	8.59887500
C	17.32686600	32.78649700	11.65037100
H	17.95015600	32.55337100	10.77428800
H	16.48539200	33.40216500	11.30038400
H	14.88739700	31.80008600	7.41370600
H	9.98901000	34.57591200	9.52098600
H	14.73286100	35.74418400	14.72416500
H	17.91821000	33.40220900	12.34432100
O	11.89995600	27.71446500	14.15233300
O	12.31437500	28.54934700	13.39705400

References

1. Ihm, C.-S.; Cho, S. J.; Paek, K., Revisiting the Water Binding of Small Cavitands: The Role of Benzene Hydrogen Bonding. *Bull. Korean Chem. Soc.* **2007,** *28* (10), 1867-1870.
2. Wu, R.; Al-Azemi, T. F.; Bisht, K. S., Influence of a resorcin[4]arene core structure on the spatial directionality of multi-arm poly(ε-caprolactone)s. *RSC Adv.* **2014,** *4* (32), 16864-16870.
3. Spek, A., Single-crystal structure validation with the program PLATON. *J. Appl. Cryst.* **2003,** *36* (1), 7-13.
4. Spek, A., Structure validation in chemical crystallography. *Acta Cryst. D* **2009,** *65* (2), 148-155.
5. Sheldrick, G. M., SHELXT - Integrated space-group and crystal-structure determination. *Acta Cryst. A* **2015,** *71*, 3-8.
6. Sheldrick, G. M., Crystal structure refinement with SHELXL. *Acta Cryst. C* **2015,** *71*, 3-8.
7. Dolomanov, O. V.; Bourhis, L. J.; Gildea, R. J.; Howard, J. A. K.; Puschmann, H., OLEX2: a complete structure solution, refinement and analysis program. *J. Appl. Cryst.* **2009,** *42*, 339-341.
8. Agresti, A.; Bacci, M.; Cecconi, F.; Ghilardi, C. A.; Midollini, S., Transition-Metal Complexes with Sulfur-Atoms as Ligands .7. Synthesis, Properties, Structure, and Molecular-Orbital Calculations of the Paramagnetic Cluster $[Fe_6(\mu_3\text{-}S)_8(PEt_3)_6](BPh_4)_2$. *Inorg. Chem.* **1985,** *24* (5), 689-695.
9. Timmerman, P.; Verboom, W.; Reinhoudt, D. N., Resorcinarenes. *Tetrahedron* **1996,** *52* (8), 2663-2704.
10. Osei, M. K.; Mirzaei, S.; Bogetti, X.; Castro, E.; Rahman, M. A.; Saxena, S.; Hernández Sánchez, R., Synthesis of Square Planar Cu_4 Clusters. *Angew. Chem. Int. Ed.* **2022,** *61* (41), e202209529.
11. Frisch, M. J.; Trucks, G. W.; Schlegel, H. B.; Scuseria, G. E.; Robb, M. A.; Cheeseman, J. R.; Scalmani, G.; Barone, V.; Petersson, G. A.; Nakatsuji, H.; Li, X.; Caricato, M.; Marenich, A. V.; Bloino, J.; Janesko, B. G.; Gomperts, R.; Mennucci, B.; Hratchian, H. P.; Ortiz, J. V.; Izmaylov, A. F.; Sonnenberg, J. L.; Williams; Ding, F.; Lipparini, F.; Egidi, F.; Goings, J.; Peng, B.; Petrone, A.; Henderson, T.; Ranasinghe, D.; Zakrzewski, V. G.; Gao, J.; Rega, N.; Zheng, G.;

Liang, W.; Hada, M.; Ehara, M.; Toyota, K.; Fukuda, R.; Hasegawa, J.; Ishida, M.; Nakajima, T.; Honda, Y.; Kitao, O.; Nakai, H.; Vreven, T.; Throssell, K.; Montgomery Jr., J. A.; Peralta, J. E.; Ogliaro, F.; Bearpark, M. J.; Heyd, J. J.; Brothers, E. N.; Kudin, K. N.; Staroverov, V. N.; Keith, T. A.; Kobayashi, R.; Normand, J.; Raghavachari, K.; Rendell, A. P.; Burant, J. C.; Iyengar, S. S.; Tomasi, J.; Cossi, M.; Millam, J. M.; Klene, M.; Adamo, C.; Cammi, R.; Ochterski, J. W.; Martin, R. L.; Morokuma, K.; Farkas, O.; Foresman, J. B.; Fox, D. J. *Gaussian 16 Rev. C.01*, Wallingford, CT, 2016.

12. Becke, A. D., Density-functional exchange-energy approximation with correct asymptotic behavior. *Phys. Rev. A Gen. Phys.* **1988,** *38* (6), 3098-3100.

13. Grimme, S.; Ehrlich, S.; Goerigk, L., Effect of the damping function in dispersion corrected density functional theory. *J. Comput. Chem.* **2011,** *32* (7), 1456-1465.

14. Weigend, F.; Ahlrichs, R., Balanced basis sets of split valence, triple zeta valence and quadruple zeta valence quality for H to Rn: Design and assessment of accuracy. *Phys. Chem. Chem. Phys.* **2005,** *7* (18), 3297-305.

15. Cancès, E.; Mennucci, B.; Tomasi, J., A new integral equation formalism for the polarizable continuum model: Theoretical background and applications to isotropic and anisotropic dielectrics. *J. Chem. Phys.* **1997,** *107* (8), 3032-3041.

16. Wolinski, K.; Hinton, J. F.; Pulay, P., Efficient implementation of the gauge-independent atomic orbital method for NMR chemical shift calculations. *J. Am. Chem. Soc.* **1990,** *112* (23), 8251-8260.

17. Lu, T.; Chen, Q., Independent gradient model based on Hirshfeld partition: A new method for visual study of interactions in chemical systems. *J. Comput. Chem.* **2022,** *43* (8), 539-555.

18. Humphrey, W.; Dalke, A.; Schulten, K., VMD: Visual molecular dynamics. *J. Mol. Graph.* **1996,** *14* (1), 33-38.

19. Lu, T.; Chen, F., Multiwfn: A multifunctional wavefunction analyzer. *J. Comput. Chem.* **2012,** *33* (5), 580-592.

20. Hancock, L. M.; McGarvey, D. J.; Plana, D., An Investigation of the Temperature Dependence of a Monomer–Dimer Equilibrium Using UV–Vis and 1H NMR Spectroscopies. *J. Chem. Educ.* **2023,** *100* (3), 1283-1288.
